# Supplementary material for: Modification of BRCA1-associated breast cancer risk by HMMR overexpression
Source: Nat Commun. 2022 Apr 7;13:1895. doi: 10.1038/s41467-022-29335-z (PMC8989921; doi:10.1038/s41467-022-29335-z)
Supplement: Supplementary file 1 — Supplementary Information [file 41467_2022_29335_MOESM1_ESM.pdf]

Modification of *BRCA1*-associated breast cancer risk by HMMR overexpression

Francesca Mateo, Zhengcheng He, Lin Mei et al.

Supplementary figure legends

Supplementary Figure 1. rs299290 is an *HMMR* eQTL in normal breast tissue and cancer. **a**, Forest plot showing the normalized effect size (NES) and 95% confidence interval (CI) of the association between rs299290-C and *HMMR* expression across human tissue in the GTEx database. The result for breast tissue is marked in red and corresponds to rs299290-C *HMMR* overexpression with nominal $p = 0.009$. **b**, Violin plots of *HMMR* expression in TCGA breast cancer subtypes and grouped by the rs299290 genotype. In the box plots inside violin plots the horizontal lines represent the sample medians, the boxes extend from first to third quartile, and the whiskers indicate values at 1.5 times the interquartile range. The number of tumors of each genotype is indicated.

Supplementary Figure 1

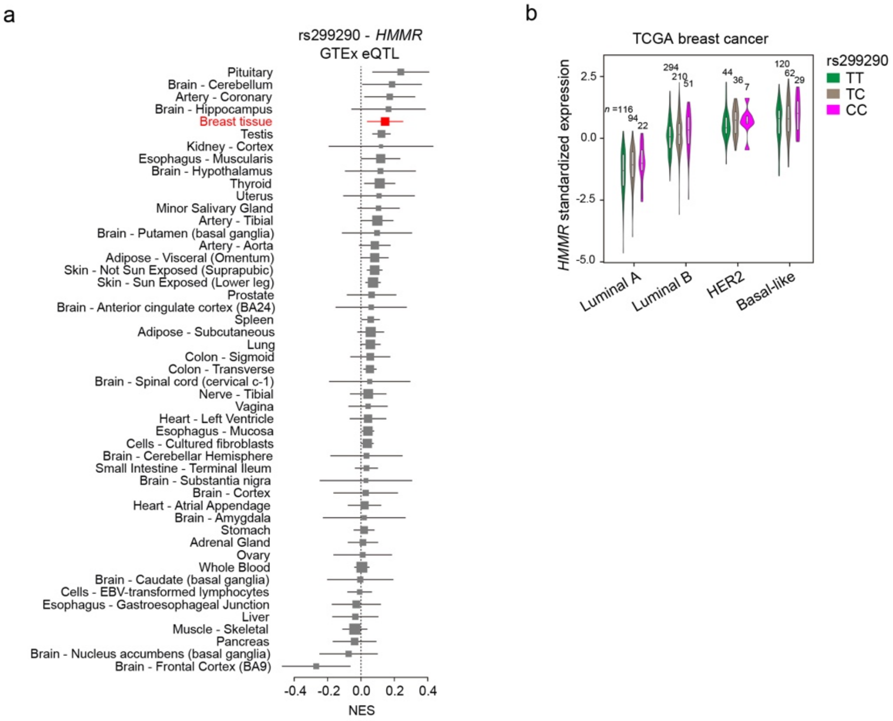

Supplementary Figure 2. rs299290 is associated with outcome of basal-like breast cancer. Kaplan–Meier plots showing progression-free interval (PFI) effects of rs299290 genotypes in breast cancer subtypes (HER2, luminal A, and luminal B). The log-rank *p* value (not significant) is shown for each setting. The number of patients of each genotype/subtype is indicated by the color-coded number at risk.

Supplementary Figure 2

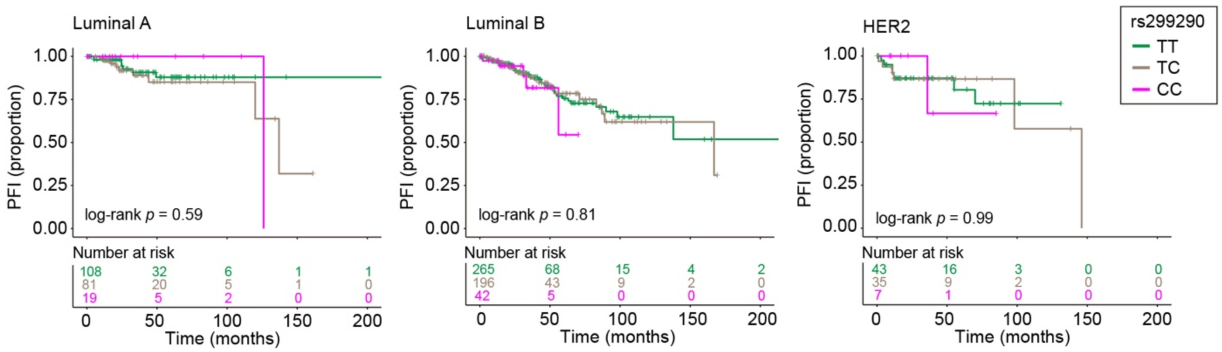

Supplementary Figure 3. rs299290 is a pan-cancer *HMMR* eQTL and is associated with features of genomic instability. rs299290 association with *HMMR* expression, homologous recombination (HR) defects, cell proliferation, aneuploidy, and fraction of genome altered. The box plots represent the sample medians, the boxes extend from first to third quartile, the whiskers indicate values at 1.5 times the interquartile range, and the outliers are shown. The median value of each feature for tumors with the rs299290-CC genotype is indicated by a red line. Pan-cancer results (left) and detailed TCGA cancer type results (right) are shown. The one-way ANOVA *p* values of each pan-cancer analysis and of each TCGA cancer result, and the number of tumors of each genotype are also shown in each panel.

Supplementary Figure 3

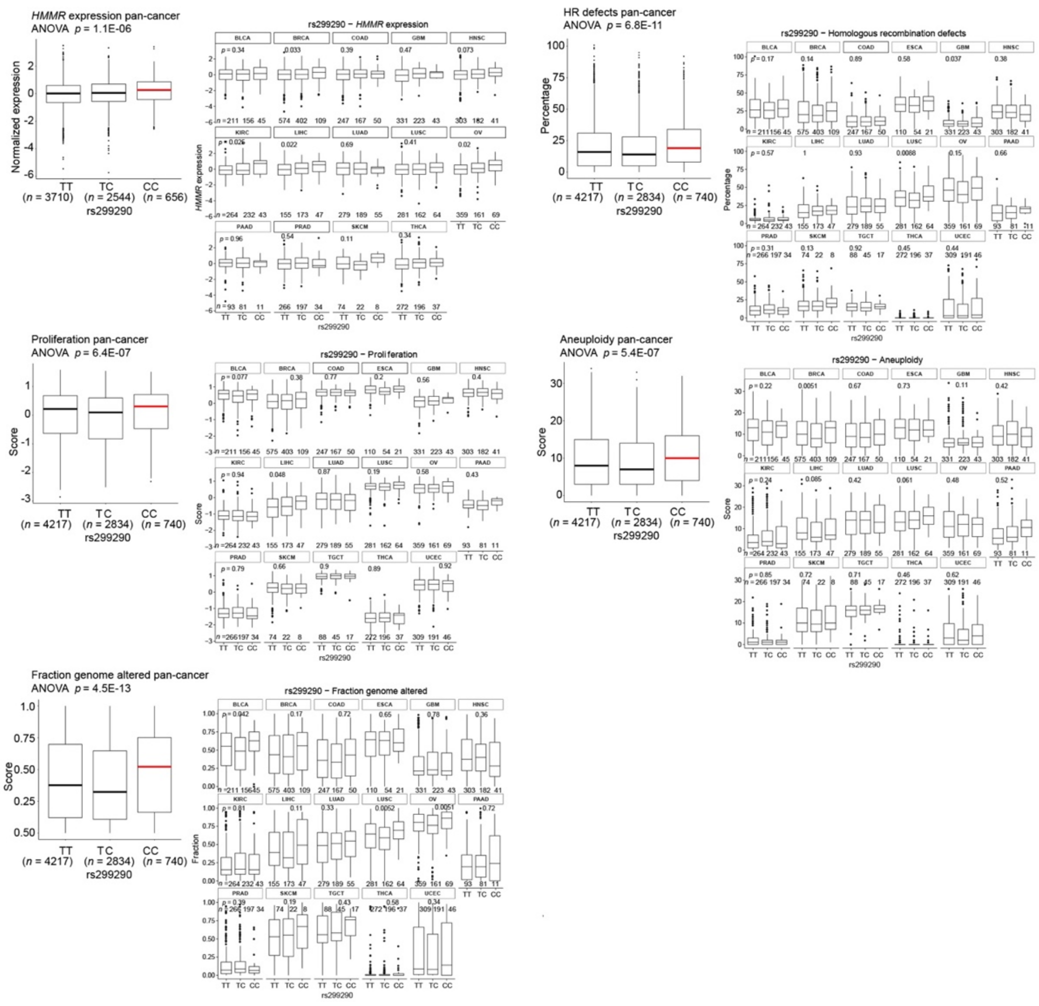

Supplementary Figure 4. Cre-induced HMMR overexpression in cells with the *loxP-STOP-loxP-HMMR* transgene. **a**, PCR detection of Cre-mediated recombination of the *loxP*-STOP-*loxP* *HMMR* cassette in mammary glands of parous $HMMR^{Tg}$ heterozygous (left panels, two individuals) and homozygous (right panels, two individuals) mice. The recombination is not detected in liver samples from the same animals. M = 100 base pair (bp) DNA ladder. **b**, Western blot results of human HMMR and loading control (tubulin α) using cell extracts of primary mouse fibroblasts transduced with a Cre-expression or empty vector, and corresponding to different genotypes (no *HMMR* transgene, +/+; one allele $HMMR^{Tg}$; or two alleles $HMMR^{Tg}$). The results of cell extracts of murine 4T1 cells (negative control) and human MCF7 cells are also shown. kDa: kilodaltons. **c**, Top panel, representative immunofluorescence images of human HMMR expression and/or DAPI staining in MECs transduced with EGFP or EGFP-Cre lentivirus. Scale bars = 40 μm. Bottom panel, quantification of HMMR intensity (mean ± s.d.; $n = 2$ experiments; $n = 30$ measurements; arbitrary units (arb. units). One-way ANOVA; ****$p < 0.0001$. **d**, Top panel, representative immunofluorescence images of mouse BRCA1 in MECs transduced with EGFP or EGFP-Cre lentivirus. Colonies were irradiated with 1 gray (Gy) to induce BRCA1-positive foci. Scale bars = 40 μm. Bottom panel, quantification of BRCA1 intensity (mean ± s.d.; $n = 2$ experiments; $n = 30$ measurements; arb. units). One-way ANOVA; ****$p < 0.0001$.

Supplementary Figure 4

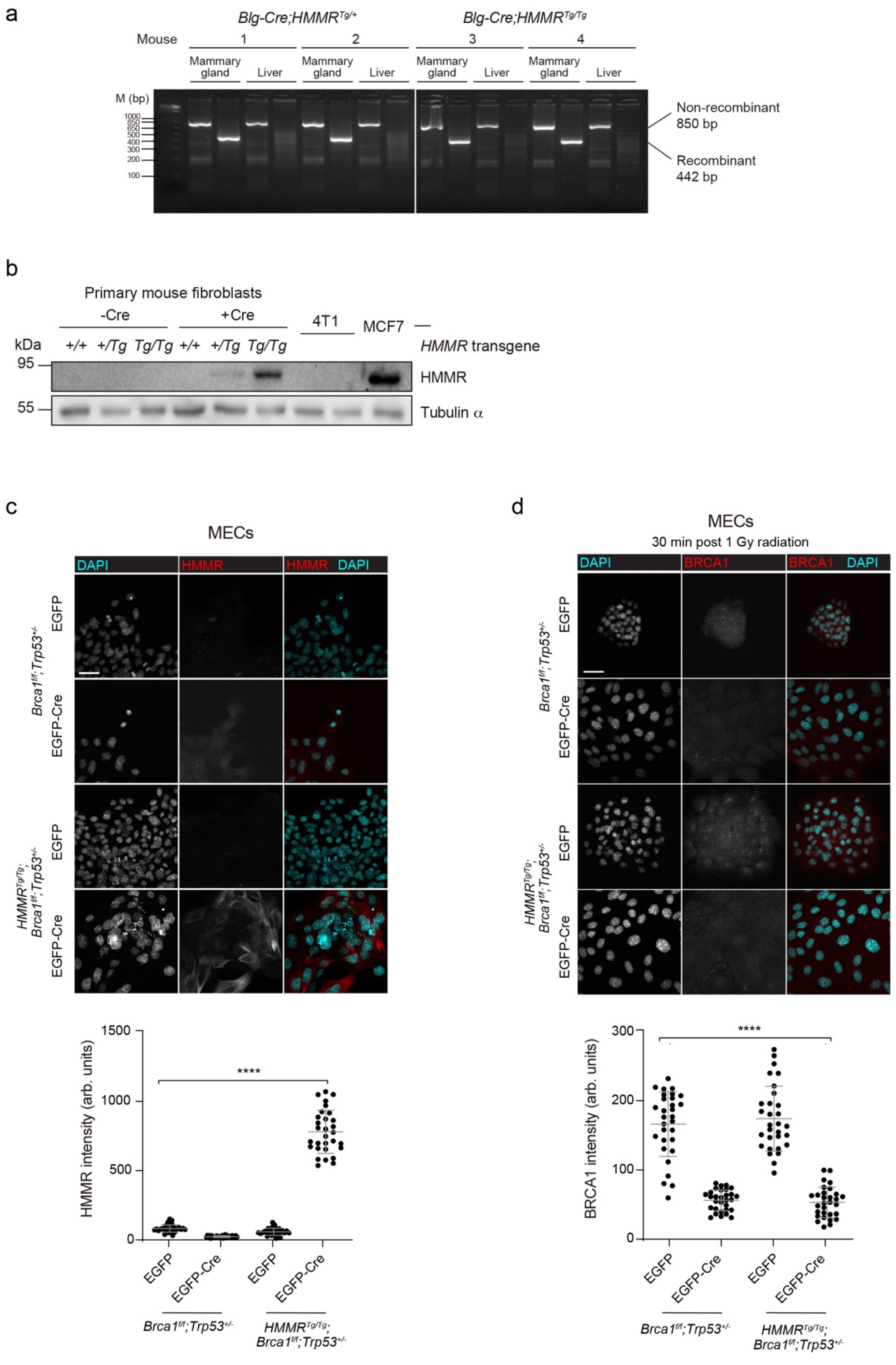

Supplementary Figure 5. No evidence of abnormal epithelial cell structures in normal mammary tissue of virgin and parous *Blg-Cre*;*HMMR*$^{Tg/Tg}$. Representative mammary gland tissue images of four animals of each genotype, at the end of the study. Scale bar = 100 µm.

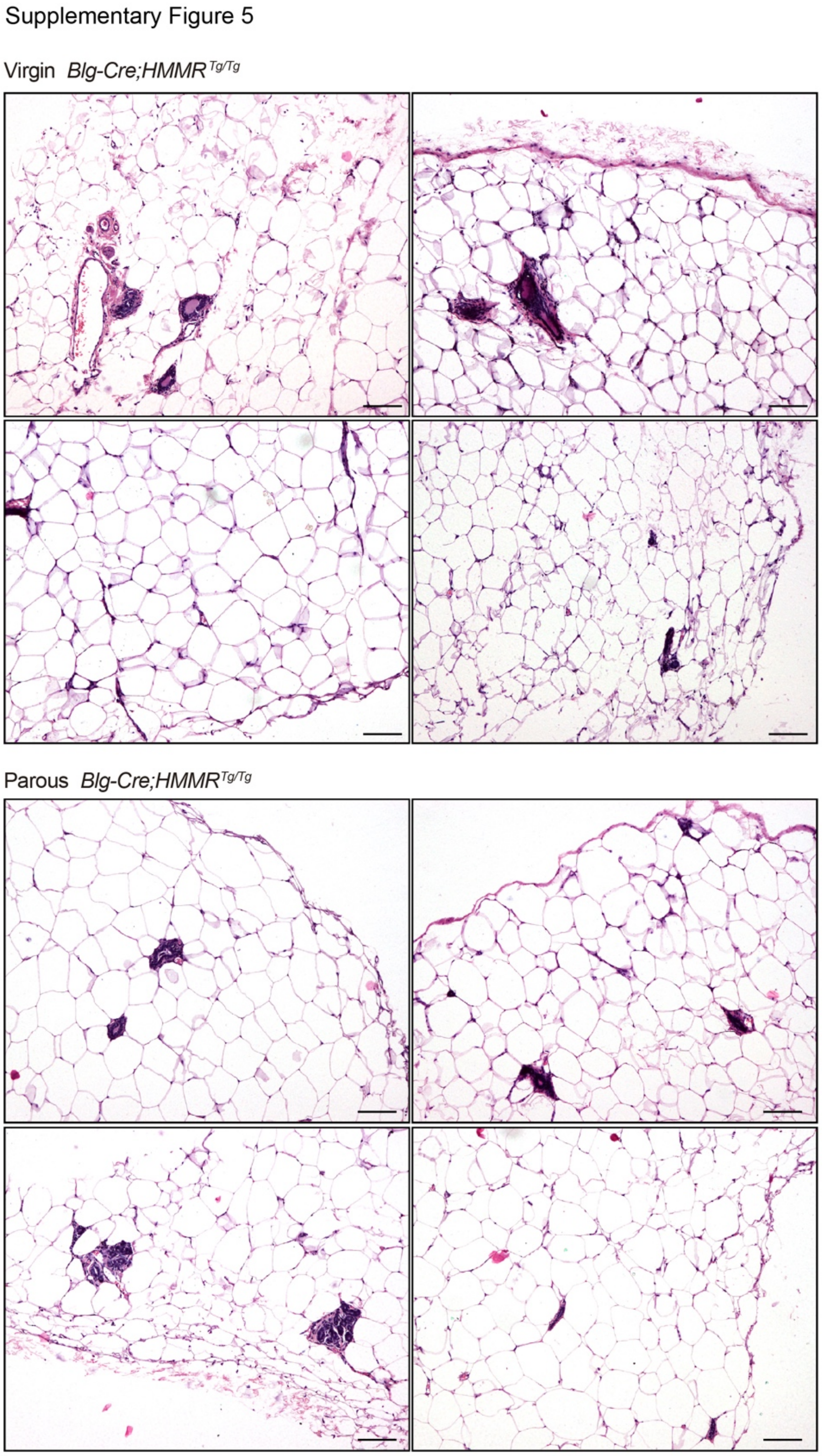

Supplementary Figure 6. Genotyping of *HMMR*, *Brca1* and *Trp53* alleles. Representative results of germline mouse DNA genotyping using targeted PCR assays. The PCR products are listed and the inferred genotypes are detailed at the bottom. M = 100 bp DNA ladder.

Supplementary Figure 6

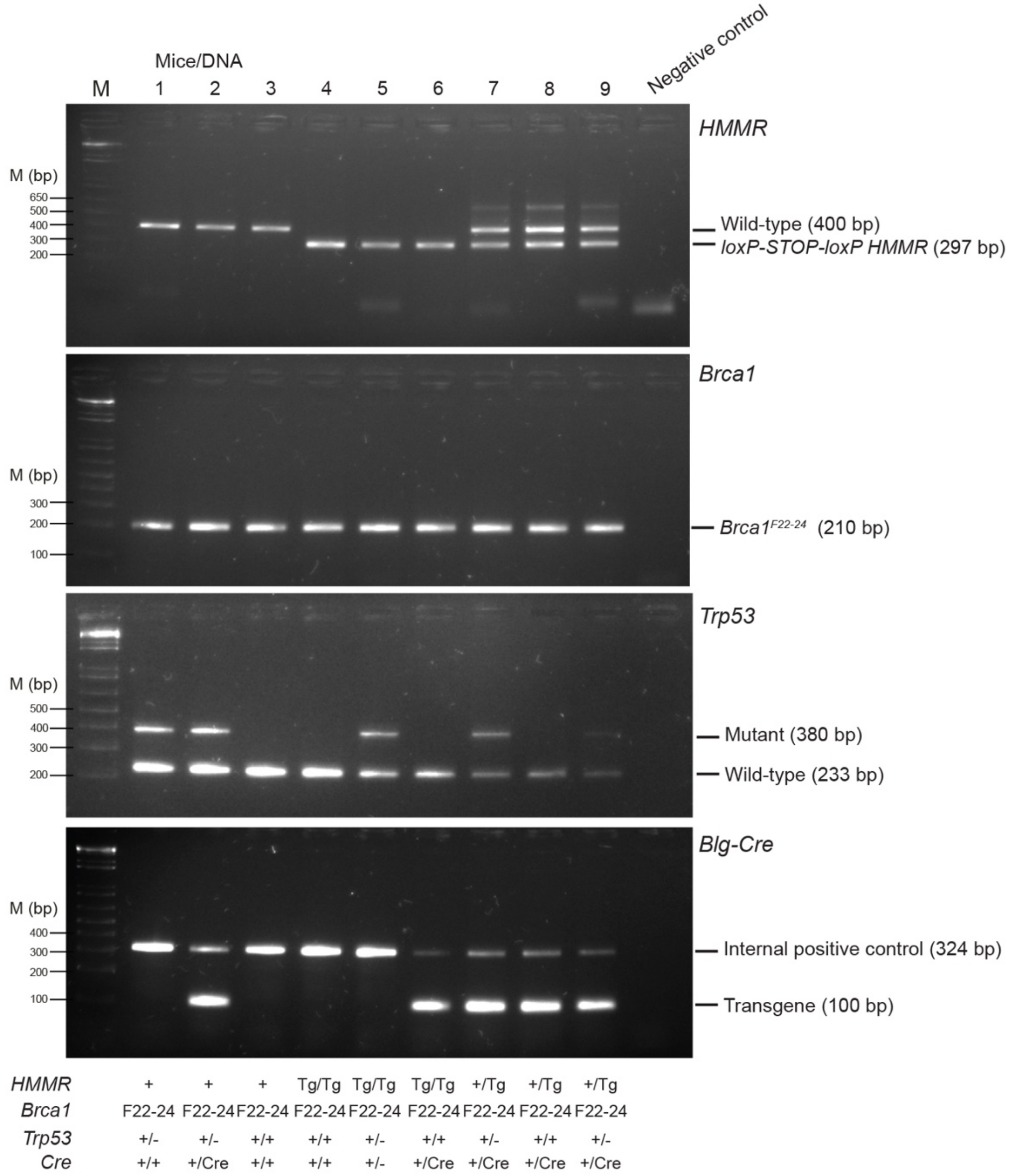

Supplementary Figure 7. Analysis of *HMMR*, *Brca1* and *Trp53* gene expression in incident tumors. Top panel, detection of *HMMR* expression by RT-PCRs using mRNA extracted from *Blg-Cre*;*HMMR*$^{Tg/Tg}$;*Brca1*$^{f/f}$;*Trp53*$^{+/-}$ tumors (right five lanes), but not in *Blg-Cre*;*Brca1*$^{f/f}$;*Trp53*$^{+/-}$ tumors (left five lanes). The mouse assay gene control is shown (*Ppia*). M = 100 bp DNA ladder. Equivalent results were obtained using TaqMan assays: human *HMMR* Hs01063280_m1 and mouse *Gapdh* Mm99999915_g1 as control. Bottom panels, expression level of *Brca1* (left) and *Trp53* (right) in mammary tumors relative to spleen and liver, respectively. The box plots show the sample medians, the boxes extend from first to third quartile, and the whiskers extend from the minimum to the maximum values (experiments *n* = 2; tumors *n* = 5 and 3 replicates; control tissue *n* = 2-3 and 3 replicates).

Supplementary Figure 7

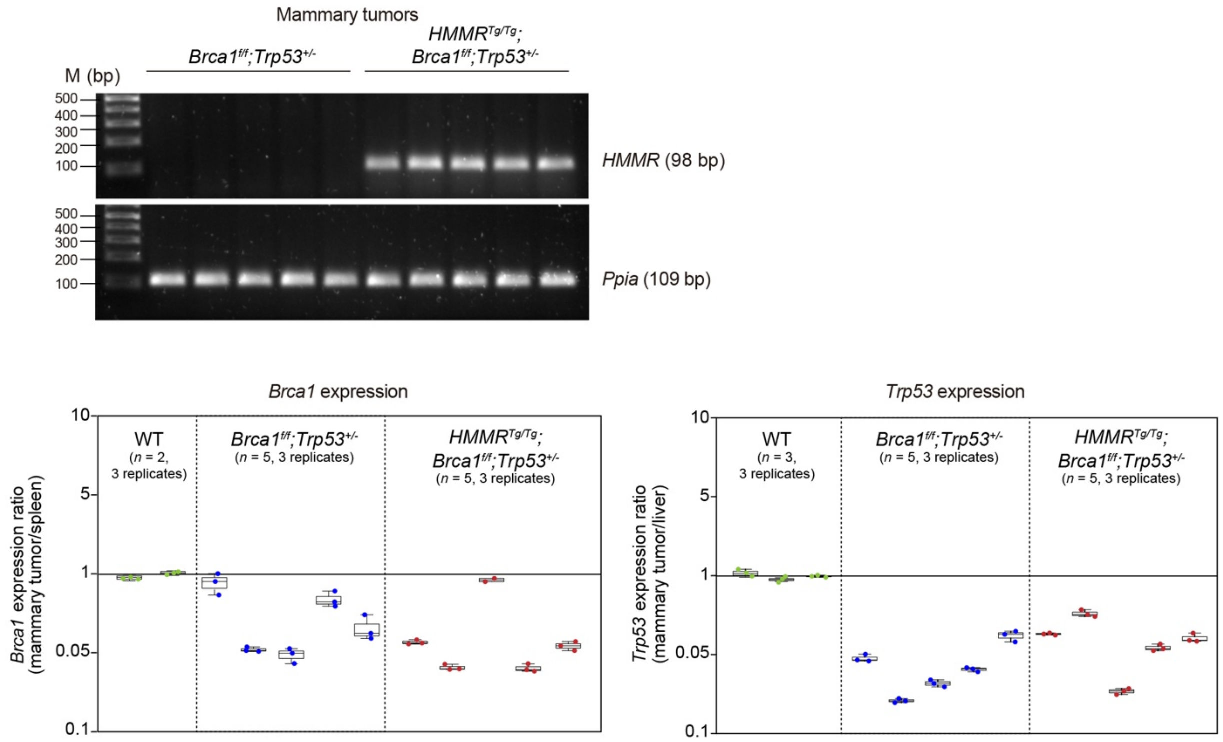

Supplementary Figure 8. Overexpression of human *HMMR* relative to endogenous expression of mouse *Hmmr*. Graphs showing the *HMMR/Hmmr* fold-change expression in tumors and mammary glands of *Blg-Cre*-induced mice with different genotypes, as shown in the inset. Two different *HMMR* Taqman probes were used relative to a single *Hmmr* probe (top of each panel). The horizontal black lines depict sample medians (*n* = 2 experiments; 3 replicates/sample).

Supplementary Figure 8

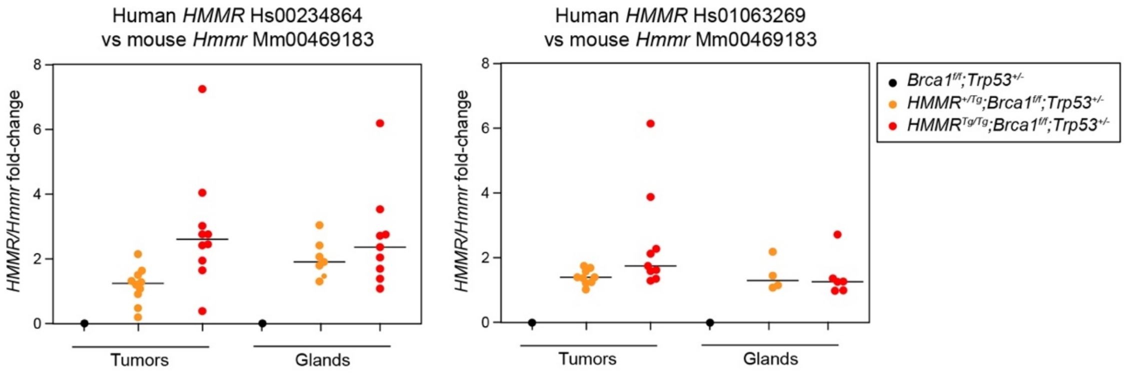

Supplementary Figure 9. Histological features of tumors. Representative images (40x magnification) of hematoxylin-eosin stained tumors, including different morphologies and features. Scale bar = 100 μm.

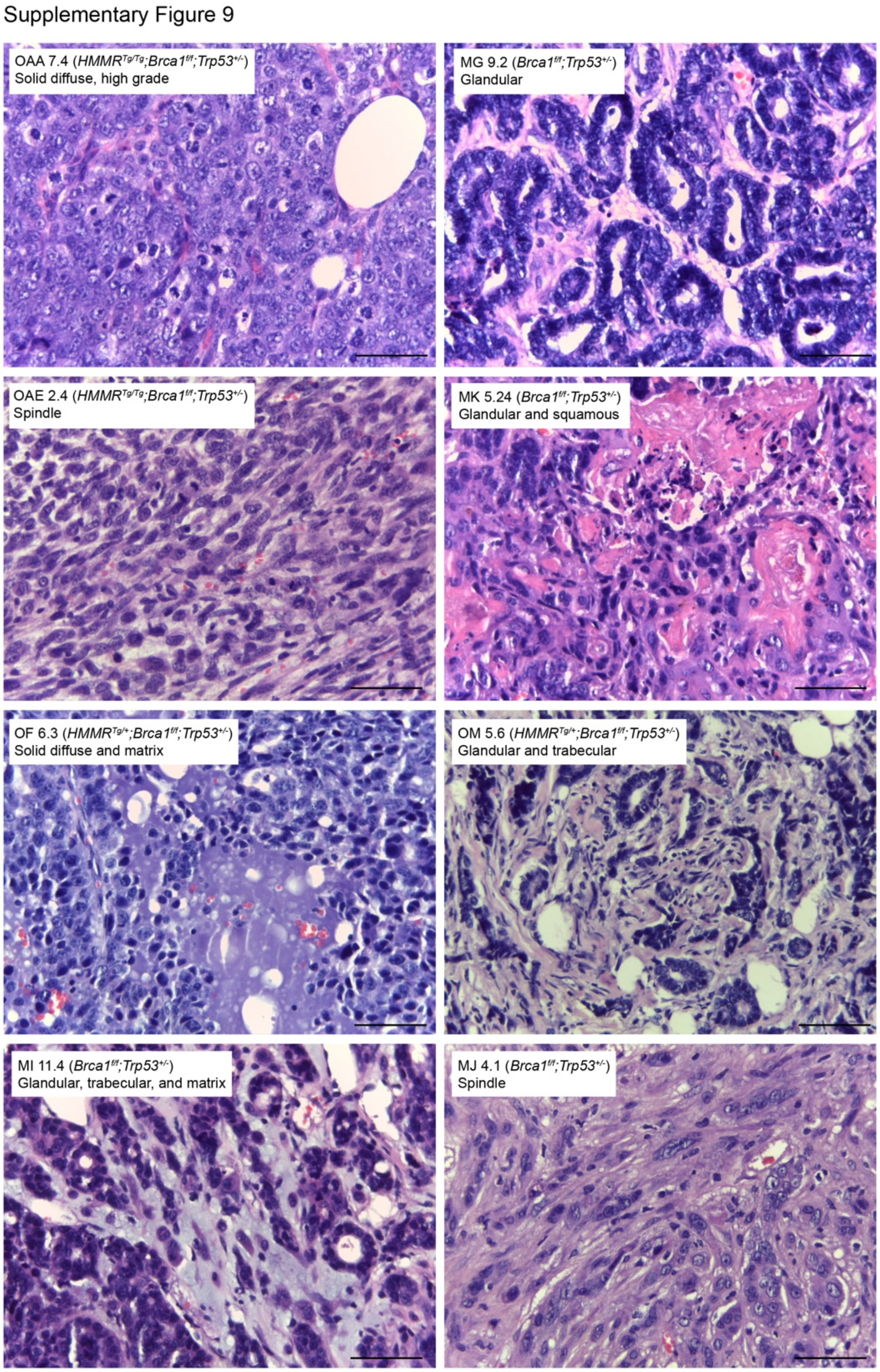

Supplementary Figure 10. Immunohistochemical study of defined tumor markers. Representative images (20X magnification) of ERα negativity (top panel; normal acini positive cells are shown in the inset), and K8/K14 negativity/positivity (middle and bottom panels). Scale bar = 100 μm.

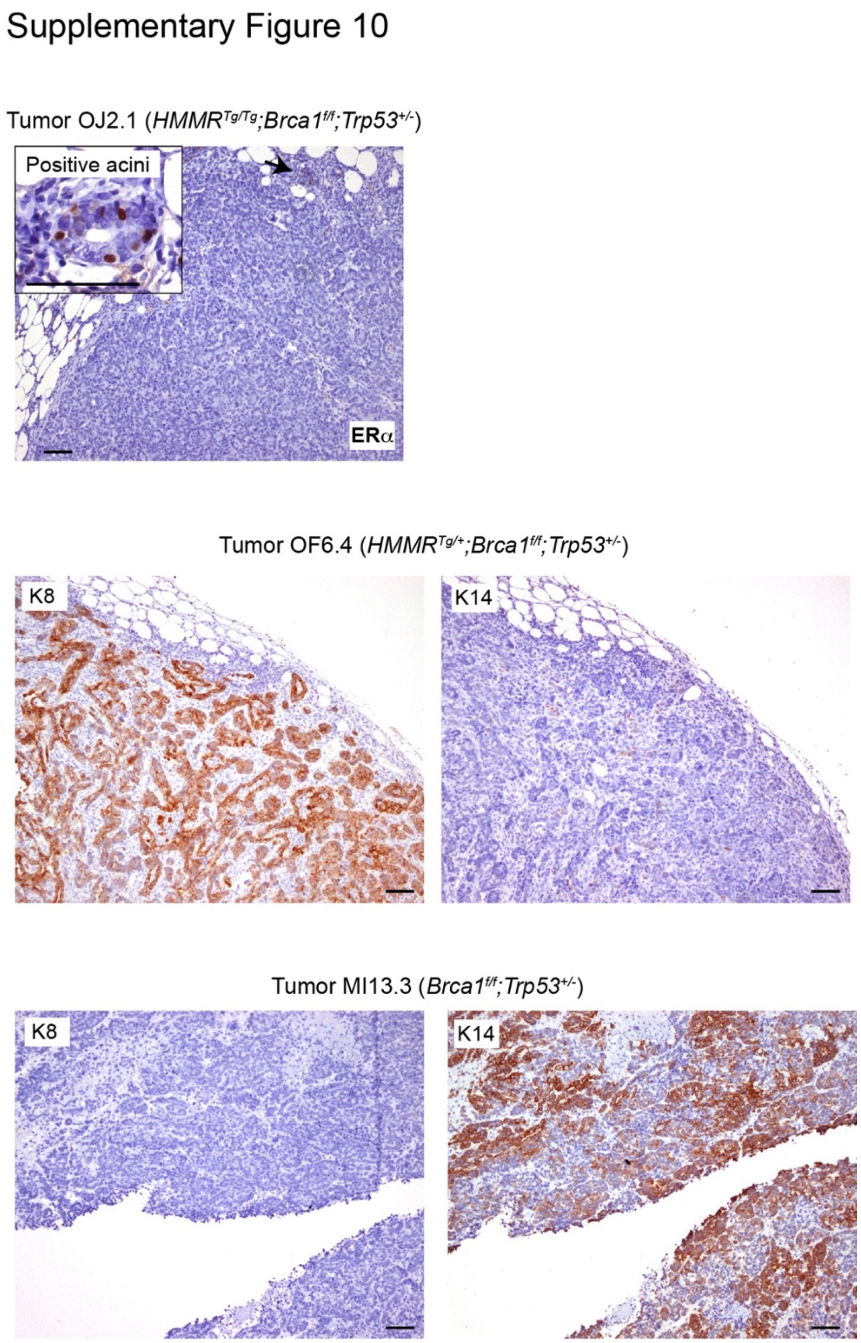

Supplementary Figure 11. *HMMR$^{Tg/Tg}$;Brca1$^{f/f}$;Trp53$^{+/-}$* tumors are associated with the triple-negative immunomodulatory and mesenchymal-like subtypes. Outputs of the GSEA tool applied with standard parameters and using the pre-ranked expression differences (RNA-seq $\log_2$-fold change) between four *HMMR$^{Tg/Tg}$;Brca1$^{f/f}$;Trp53$^{+/-}$* and four *Brca1$^{f/f}$;Trp53$^{+/-}$* tumors, and gene sets corresponding to upregulated genes originally identified in the six Lehman's subtypes (basal-like 1 (BL1), BL2, immunomodulatory (IM), luminal-androgen receptor (LAR), mesenchymal (M), and mesenchymal-like (ML)). The GSEA NES and p value are shown for each gene set analysis (not significant, n.s.). The red rectangles indicate the over-expressed leading edges of IM and ML genes in *HMMR$^{Tg/Tg}$;Brca1$^{f/f}$;Trp53$^{+/-}$* tumors.

Supplementary Figure 11

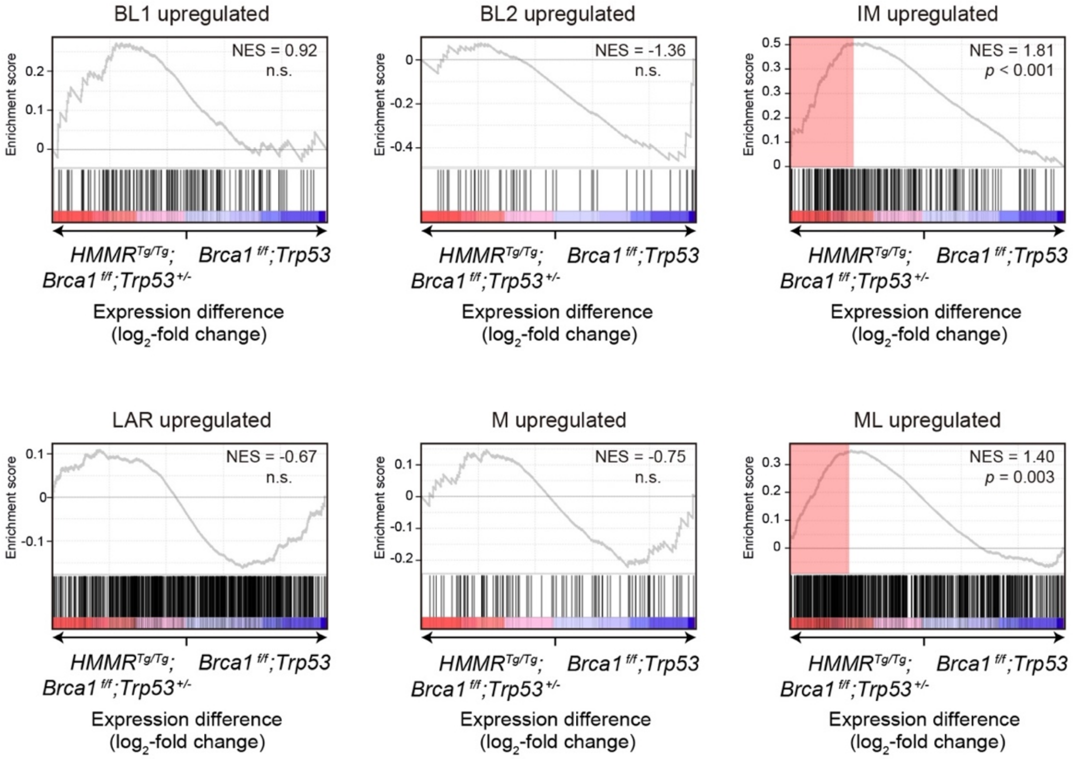

Supplementary Figure 12. Measurement of phenotype of MEC CFC colonies. **a**, Example of a colony derived from primary mouse MECs that displays an epithelial phenotype. The boxed area (100 x 100 μm^2) was used to determine the density of nuclei in the colony. Colonies with a nucleus density > 40 were classified as epithelial; those with a nucleus density < 20 were classified as mesenchymal or EMT. Scale bar = 40 μm. **b**, Frequency of nuclei per box in colonies with epithelial (n = 9) or mesenchymal (n = 9) phenotypes (mean ± s.d.; n = 3 experiments; n = 3 colonies/experiment). Two-sided Student's unpaired-samples t-test; ****$p < 0.0001$. **c**, Expression of CLDN1, VIM, and ZO-1 in MEC colonies with epithelial or EMT phenotype, as determined by the density of nuclei. Scale bars = 40 μm. **d**, Immunofluorescence detection of LMNB1 in subconfluent MEC cultures transduced with EGFP-only or EGFP-Cre lentivirus and with defined genotypes. Zoomed images are taken from Fig. 4b. Scale bars = 10 μm.

Supplementary Figure 12

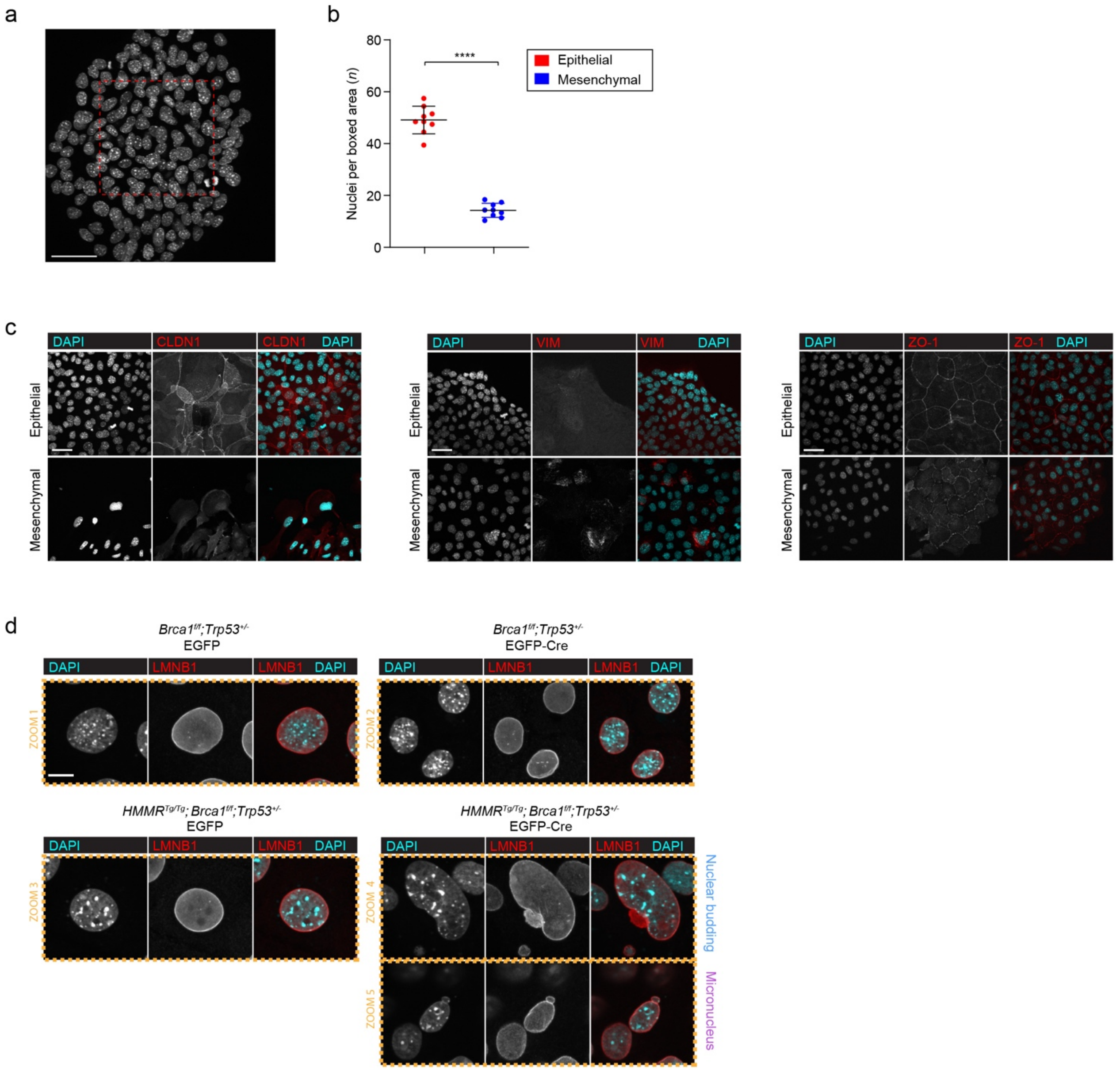

Supplementary Figure 13. HMMR overexpression is associated NF-κB signaling. **a**, Immunofluorescence analysis of the localization and levels of p65 in day 5 colonies generated from MECs cultures with indicated genotypes following transduction with indicated lentivirus (EGFP-empty or EGFP-Cre). Scale bars = 20 μm. **b**, Immunofluorescence analysis of the localization and levels of p52 in day 5 colonies generated from MECs with indicated genotypes following transduction with indicated lentivirus. Scale bars = 20 μm.

Supplementary Figure 13

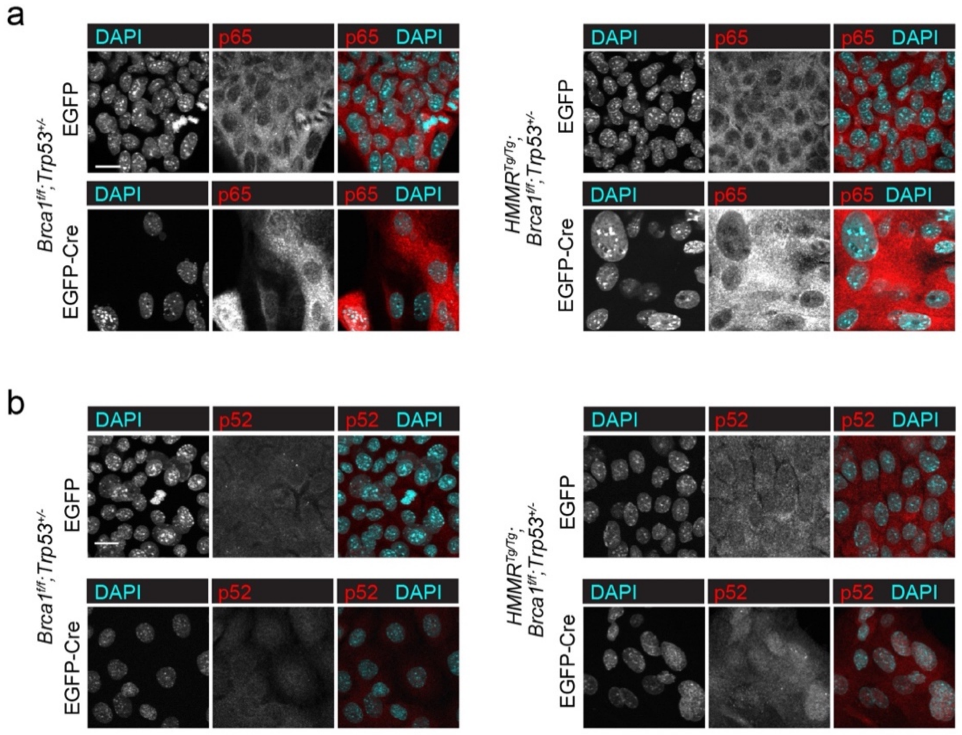

Supplementary Figure 14. *HMMR*$^{Tg/Tg}$;*Brca1*$^{f/f}$;*Trp53*$^{+/-}$ tumors are associated with a non-canonical NF-κB expression program. GSEA results using standard parameters and pre-ranked expression differences (RNA-seq $\log_2$-fold change) between four *HMMR*$^{Tg/Tg}$;*Brca1*$^{f/f}$;*Trp53*$^{+/-}$ tumors and four *Brca1*$^{f/f}$;*Trp53*$^{+/-}$ tumors, and gene sets corresponding to RELB:p52 and RELA:p50 gene targets based on conserved binding motifs (targets common to the two sets were excluded from the analysis). The GSEA NES and *p* value (Kolmogorov-Smirnov statistic and x1000 permutation test) are shown for each analysis. The red rectangle indicates the overexpressed leading edge associated with RELB:p52 targets, and the corresponding genes are depicted, including *Vim*.

Supplementary Figure 14

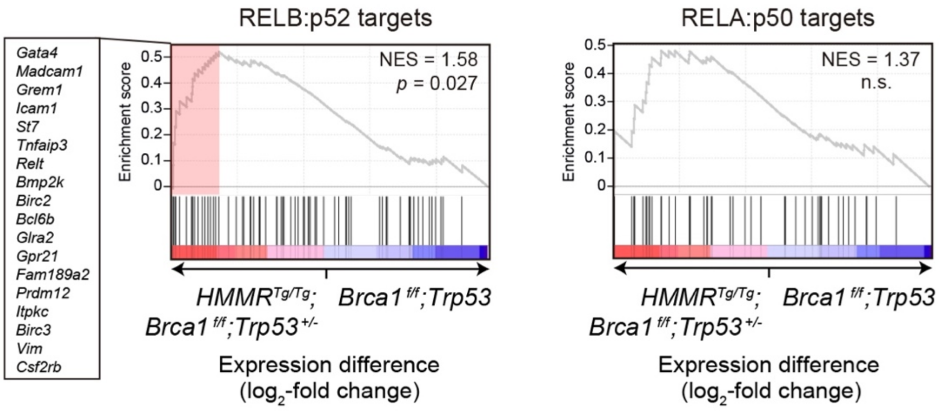

Supplementary Figure 15. GFP-HMMR expression in HeLa (Tet-On) cells delays metaphase and disturbs the intrinsic spindle positioning pathway. a, Tet-On HeLa cells were treated with water (-dox) or doxycycline (+dox) for 24 hours to induce GFP-HMMR expression. Cells were synchronized at M phase with nocodazole and MG132, lysed, and HMMR expression was quantified by western blot, followed by Licor imaging. Equal loading was verified by GAPDH level. Signal intensity (arbitrary units (arb. units)) was quantified and tabulated. kDa: kilodaltons. **b**, Left panels, images of mitotic progression of HeLa cells or Tet-On HeLa cells treated with water (-dox) or doxycycline (+dox) for 24 hours, followed by live cell imaging. Mitotic phases are color-coded and the mitotic kinetics of 50 cells per condition are tabulated (right panels). Scale bar = 5 μm. **c**, Quantification of metaphase duration of HeLa cells or Tet-On HeLa cells treated with water (-dox) or doxycycline (+dox) for 24 hours followed by live cell imaging (mean ± s.d.; n = 50 cells; n = 2 experiments; n = 25 cells per experiment). One-way ANOVA; $^{**}p = 0.003$. **d**, Illustration of spindle rotation during metaphase. Spindle orientation at the beginning of metaphase (red dashed line) and before anaphase (blue dashed line) is highlighted to demonstrate spindle rotation.

Supplementary Figure 15

a

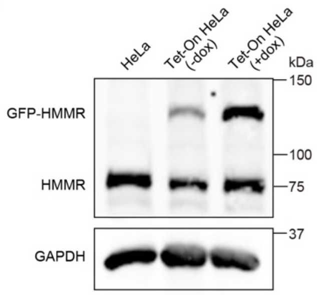

Analysis of immunoblot signal intensity (arb. units)

Protein \ Cell	HeLa	Tet-On HeLa (-dox)	Tet-On HeLa (+dox)
GFP-HMMR	n/a	56.2	163.4
HMMR	196.9	127.0	166.4
Total	196.9	183.2	329.7

b

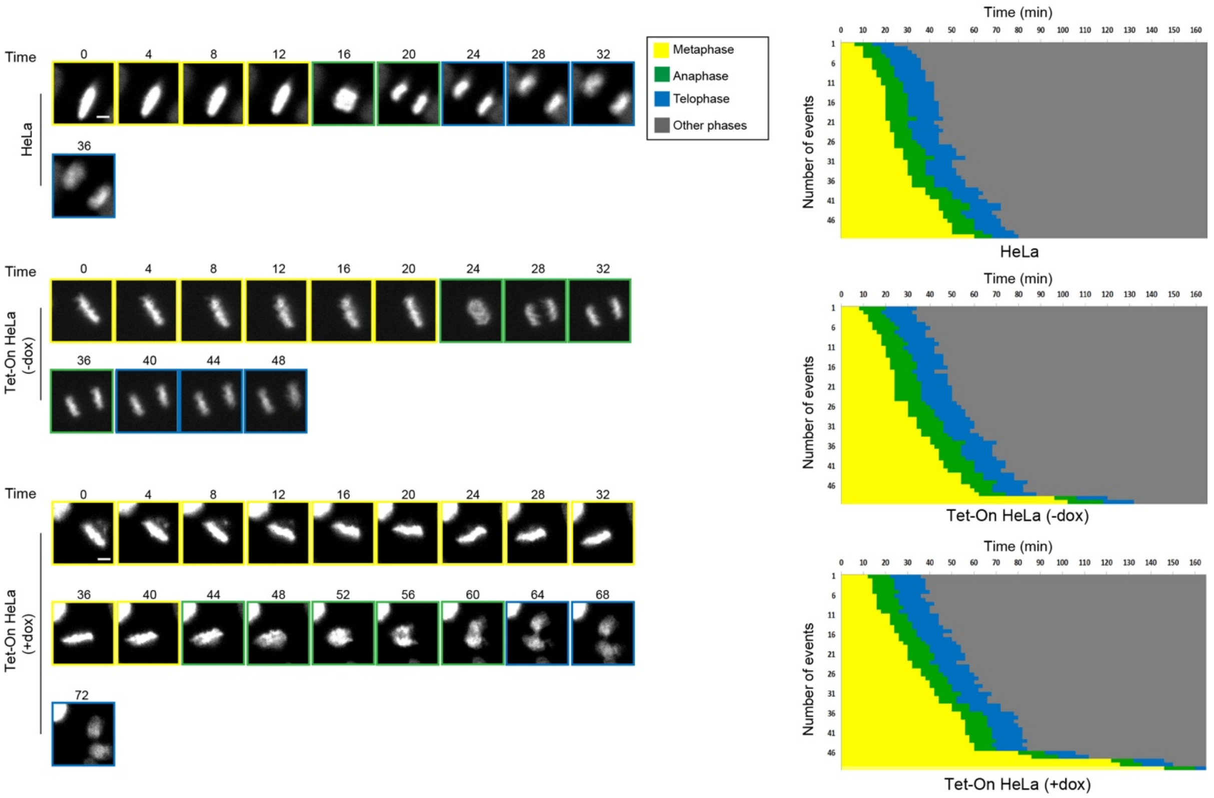

c

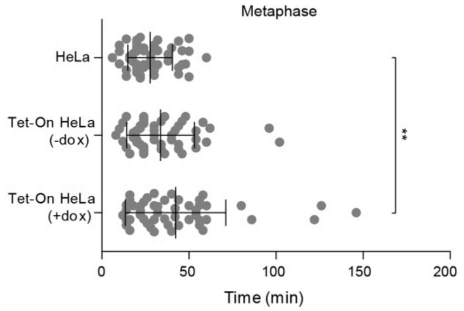

d

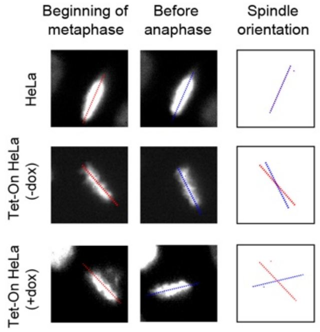

Supplementary Figure 16. GFP-HMMR overexpressing HeLa cells display abnormal chromosomes during cell division. Top panels, HeLa or Tet-On HeLa cells were incubated with CellLight Actin-RFP BacMam 2.0 for 2 days to visualize F-actin. Cells were pre-treated with water (-dox) or doxycycline (+dox) for 24 hours before imaging. Mitotic cells images were captured every 5 minutes. Yellow stars indicate membrane blebs and green arrowheads indicate the lagging chromosomes. Scale bar = 10 µm. Bottom panels indicate measurements from images of ~80 cell divisions per treatment and the resultant daughter cells, including the proportion of cells displaying anaphase membrane blebs (left panel; mean ± s.d.; n = 2 experiments; 2 wells per experiment); two-sided Student's paired-samples t-test; **p = 0.001. Daughter cell size ratio; mean ± s.d.; n = 2 experiments; n = 77 (HeLa), 80 (-dox), and 82 (+dox) mitotic cells; two-sided Student's paired-samples t-test, ***p = 0.001; bleb size mean ± s.d. from n = 19 (HeLa), 32 (-dox), 63 (+dox) blebbing anaphase cells; two-sided Student's paired-samples t-test, *p = 0.022; and abnormal chromosomes for mitotic cells and their resultant daughter cells; mean ± s.d.; n = 2 experiments; n = 77 (HeLa), 80 (-dox), and 82 (+dox) mitotic cells; one-way ANOVA; **p = 0.008.

Supplementary Figure 16

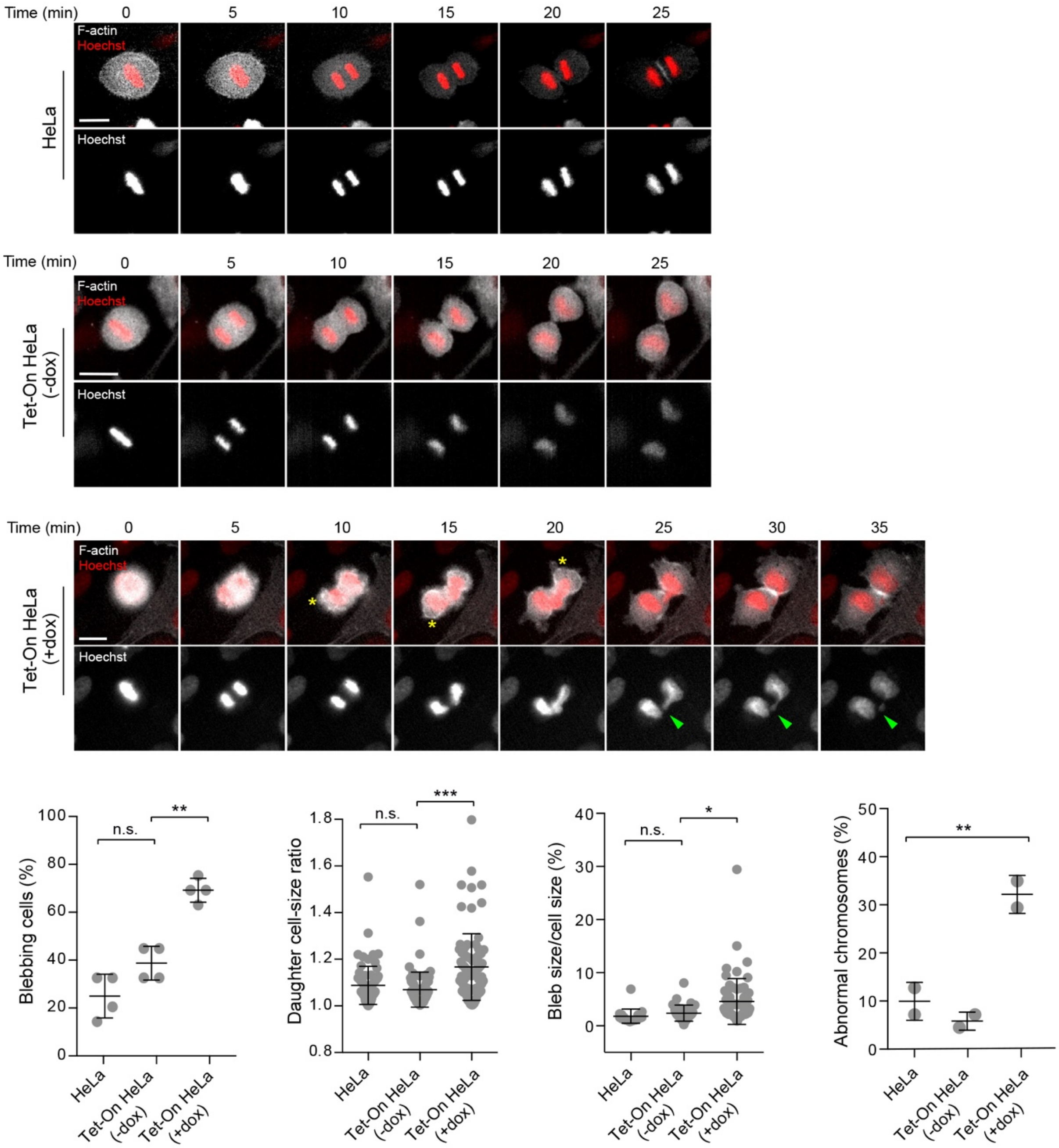

Supplementary Figure 17. HMMR interactome during mitosis. Mass spectrometry analysis of proteins co-precipitated from mitotic HeLa lysates with antibodies targeting HMMR or control immunoglobulin (log_2-fold change in HMMR:control ratio). Known HMMR-binding partners are highlighted in blue and actin-binding proteins (ARP3 (ACTR3), MYH10, and MYO18A) are highlighted in red. The plot shows the $-log_{10}$ two-sided Student's *t*-test nominal *p* values against log_2 fold-change of HMMR versus control immunoglobulin IP results. The vertical and horizontal dashed lines indicate $p < 0.05$ and log_2 fold-change > 1, respectively. The symbols indicate the frequency at which proteins were found in the interaction screens as listed in the CRAPOME database (circle: rare interactor; open circle: common interactor; diamond: not listed in database); protein names in blue, red, and grey indicate known HMMR interactors, potential interactors (this study), and additional proteins found moderately to strongly enriched, respectively; and symbol size was proportional to the number of replicas a protein was identified in ($n = 2$ experiments; 2 replicates/condition).

Supplementary Figure 17

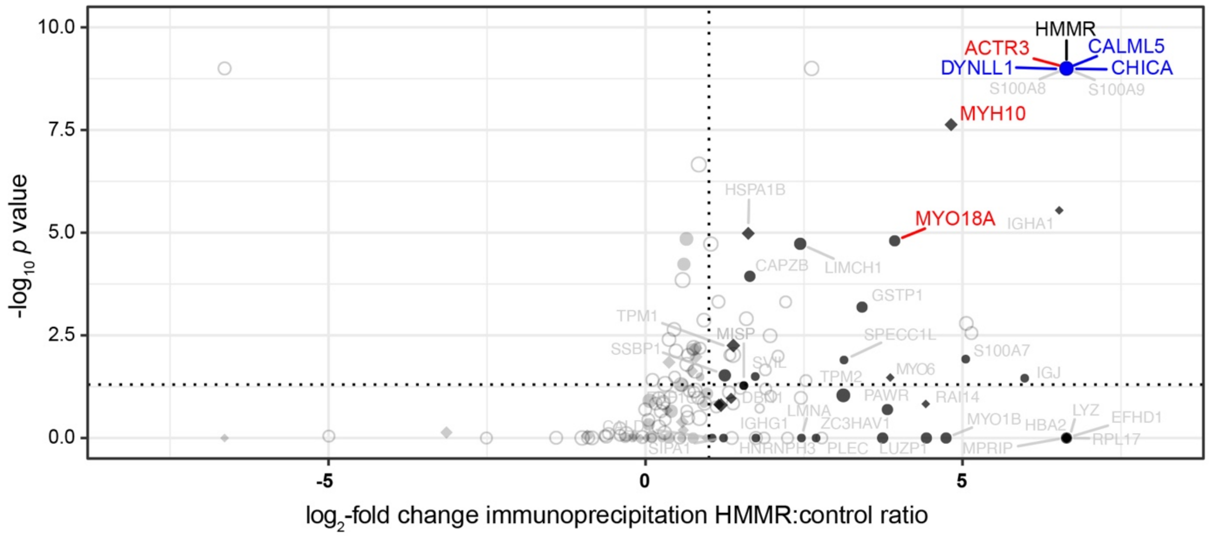

Supplementary Figure 18. Localization of non-muscle myosin MYH9 and MYH9 in GFP-HMMR (Tet-On) HeLa cells. **a**, Immunofluorescence analysis of GFP-HMMR overexpressing anaphase cells and control HeLa cells probing for HMMR and MYH9. Scale bar = 5 μm. White, pink, and red dashed lines in the middle column indicate the measurement of plot profile in panel **b**. **b**, Plot profile measuring fluorescence intensity across the anaphase cells shown by the dashed lines in panel **a**. The blue shaded areas indicate 3 μm from the cortex. Arbitrary units (arb. units). **c**, Cortical MYH9 quantification in anaphase cells. The color-coded averages of three experiments are shown; mean ± s.d.; *n* = 30 (HeLa), 30 (-dox), 30 (+dox) cells. Two-sided Student's paired-samples *t*-test; n.s. **d**, Average intensity of MYH9 in anaphase cells; mean ± s.d.; *n* = 30 (HeLa), 30 (-dox), 30 (+dox) cells. Two-sided Student's unpaired-samples *t*-test; ****p* = 0.001. **e**, Immunofluorescence analysis of GFP-HMMR overexpressing anaphase cells and control HeLa cells probing for HMMR and MYH10. Scale bar = 5 μm. White, pink, and red dashed lines in the middle column indicate the measurement of plot profile in panel **f**. **f**, Plot profile measuring fluorescence intensity across the cells shown by the dashed lines in panel **e**. The blue shaded areas mark 3 μm from the cortex. **g**, Cortical MYH10 enrichment in anaphase cells presenting the color-coded averages of three experiments; mean ± s.d.; *n* = 30 (HeLa), 30 (-dox), 30 (+dox) cells. Two-sided Student's paired-samples *t*-test; n.s. **h**, Average intensity of MYH10 in GFP-HMMR overexpressing cells and control cells; mean ± s.d.; *n* = 30 (HeLa), 30 (-dox), 30 (+dox) cells. Two-sided Student's unpaired-samples *t*-test; ***p* = 0.002; and two-sided Student's paired-samples *t*-test, **p* = 0.013.

Supplementary Figure 18

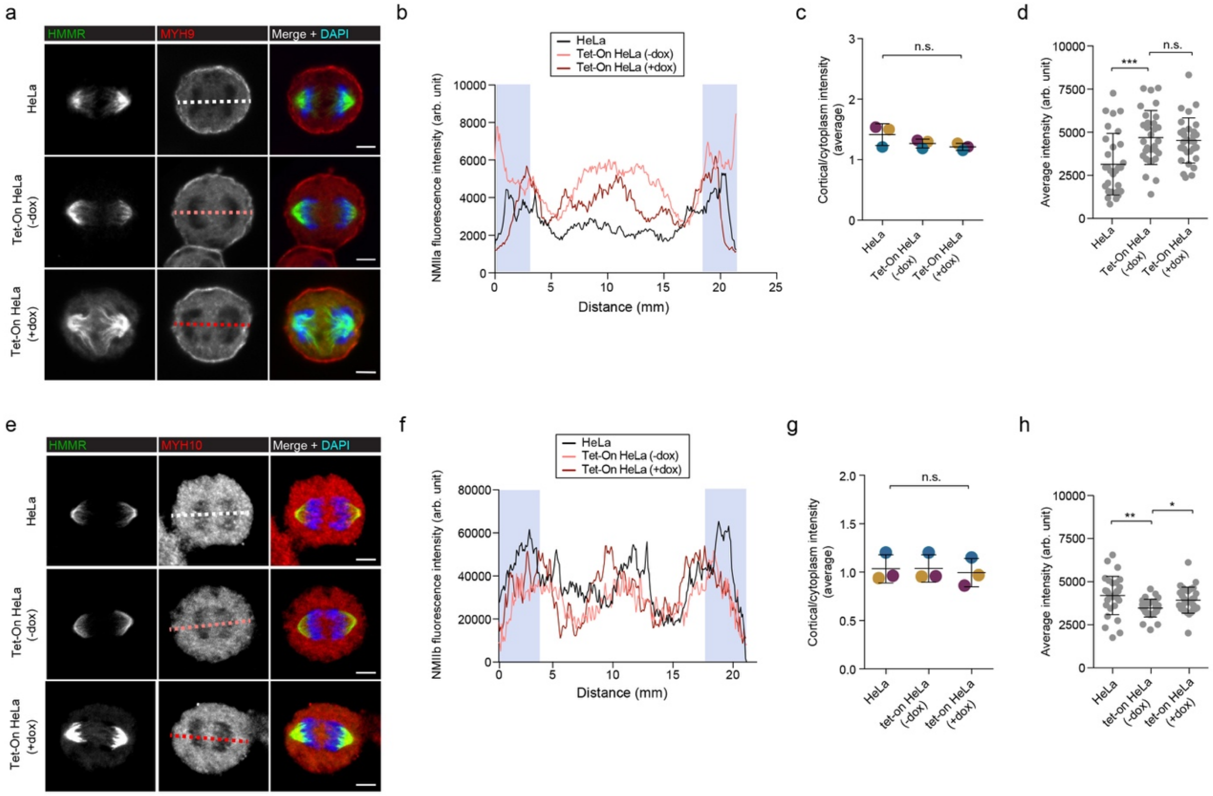

Supplementary Figure 19. ARPC2 is mislocalized in HMMR overexpressing HeLa cells during metaphase and anaphase. **a**, Immunofluorescence analysis of ARPC2 and HMMR in mitotic cells. HeLa cells were arrested at prometaphase using S-trityl-L-cysteine (STLC, 5 μM) for 16 hours and forced to exit mitosis by the addition of RO-3306 (20 μM) for 5 minutes. Scale bar = 5 μm and for zoom = 2 μm. **b**, Immunofluorescence analysis of ARPC2 and pericentrin (PCNT) in mitotic cells. HeLa cells were arrested at prometaphase (+STLC) or forced to exit mitosis (+RO-3306). Scale bar = 5 μm and for zoom = 2 μm. **c**, Spindle pole intensity for ARPC2 at prometaphase and forced mitotic exit presenting the color-coded averages of three experiments (mean ± s.d.; $n = 60$ prometaphase arrest and $n = 60$ forced mitotic exit cells). Two-sided Student's paired-samples *t*-test; $^{*}p = 0.031$. **d**, Immunofluorescence analysis of ARPC2 and pericentrin in mitotic cells overexpressing GFP-HMMR. GFP-HMMR expression induced Tet-On HeLa cells (+dox) and control (-dox) cells were arrested at prometaphase (+STLC) for 16 hours and forced to exit mitosis (+RO-3306) for 5 minutes. Scale bar = 5 μm (prometaphase arrest and forced mitotic exit +/- dox images) and 2 μm (zoom images). **e**, Spindle pole intensity of ARPC2 at prometaphase and anaphase in GFP-HMMR induced Tet-On HeLa cells (+dox) and control (-dox) cells (mean ± s.d.; 20 cells per experiment per treatment). Two-sided Student's unpaired-samples *t*-test; $^{****}p < 0.0001$.

Supplementary Figure 19

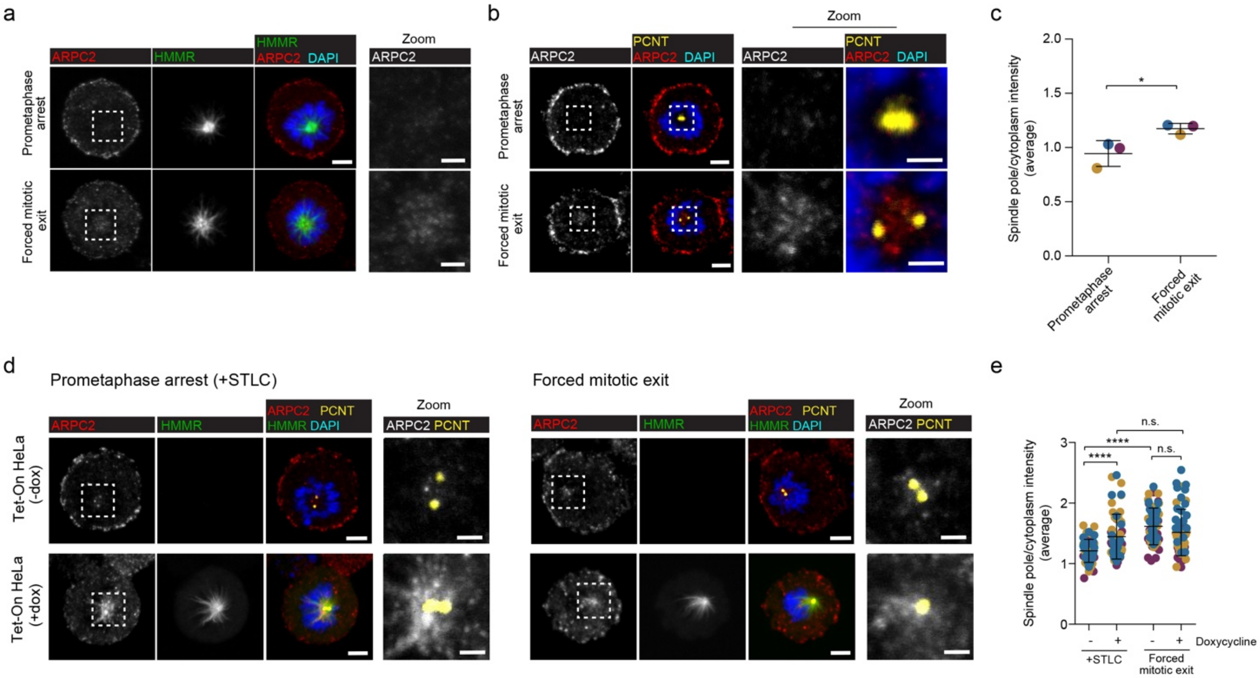

Supplementary Figure 20. Evaluation of endogenous co-immunoprecipitation of HMMR and ACTR3. Western blot results of HMMR immunoprecipitation using different methods of cell synchronization, as depicted at the bottom of each panel. While CHICA was found to co-immunoprecipitate with HMMR, no robust signal was detected for ACTR3 in these conditions. The assays also evaluated MYH10 and GAPDH (negative control). WCL: Whole cell lysate; and IP: immunoprecipitation. kDa: kilodaltons.

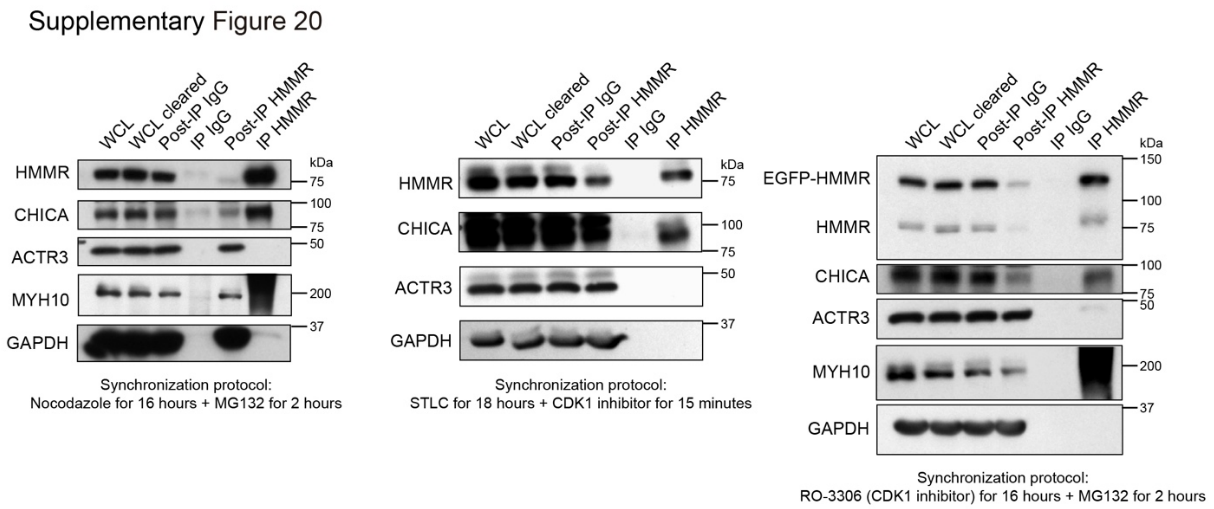

Supplementary Figure 21. HMMR overexpression is associated with abnormal earlier detection of ARPC2 at the mitotic spindle poles. **a**, Representative images of immunofluorescence detection of HMMR (EGFP-tagged), ARPC2, and β-tubulin (TUBB) in parental and Tet-On (-dox and +dox) HeLa cells through interphase/G2 and mitosis. Scale bar = 10 µm. **b**, Quantification of ARPC2 signal intensity at the centrosome (interphase/G2 phase) and spindle poles during phases of mitosis, relative to cytoplasmic intensity (mean ± s.d; n = 2 experiments for interphase-prometaphase; n = 3 experiments for metaphase-anaphase). Two-sided Student's unpaired-samples t-test; $^{**}p = 0.004$ and $^{****}p < 0.0001$.

Supplementary Figure 21

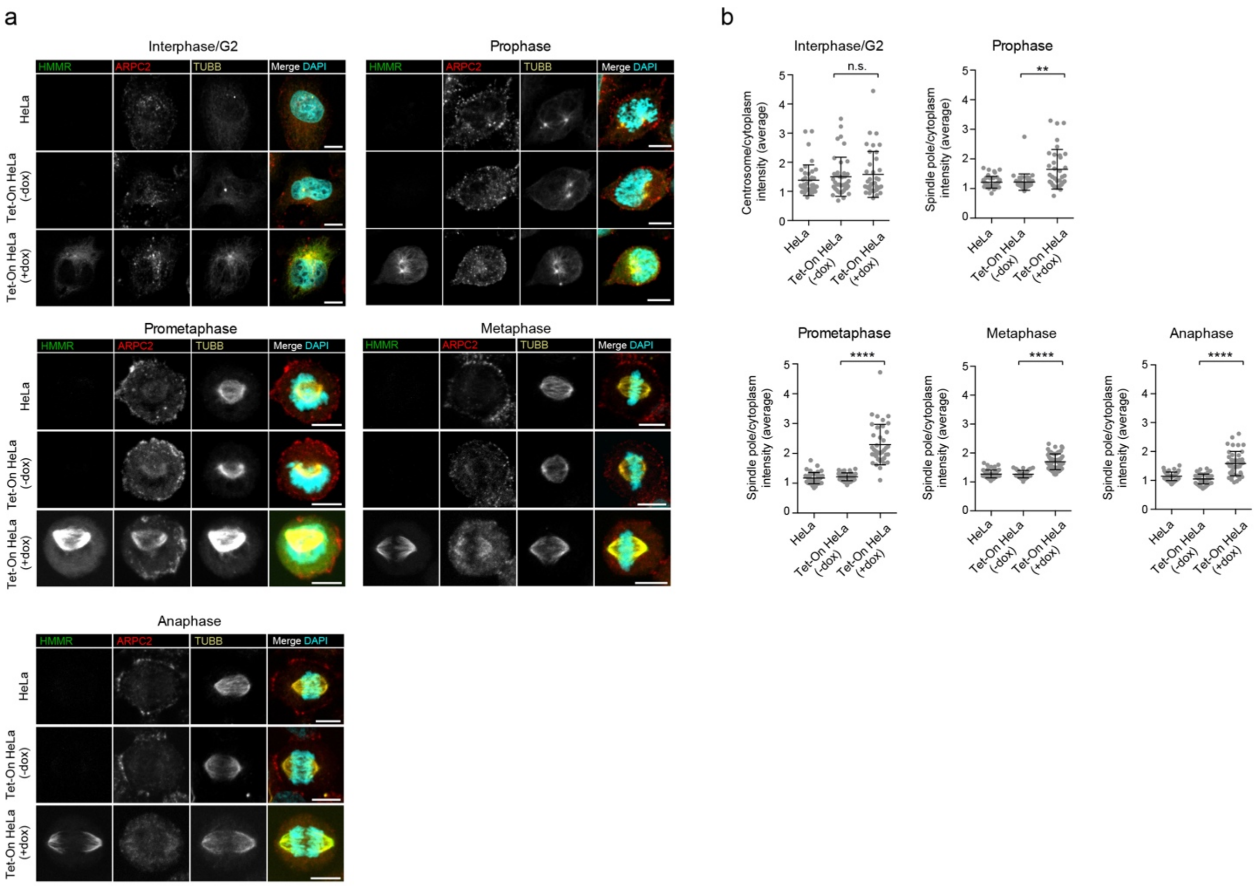

Supplementary Figure 22. AURKA inhibition normalizes blebbing and daughter cell size. a, Evaluation of viability of HeLa cells exposed to kinase inhibitors (X-axis, log M concentration). The data points of three experiments are shown. The half-maximal inhibitory concentration (IC50) of each compound is denoted at the top of each panel. The blue areas correspond to the compound concentration range used in subsequent panels ($n = 3$ experiments). **b**, Quantification of nuclear blebbing (%) of HeLa cells exposed to vehicle (DMSO), and of HMMR-overexpressing HeLa cells exposed to DMSO or increasing concentrations of each inhibitor. The AURKA inhibitor (MLN8237) significantly reduces nuclear blebbing (mean ± s.d; $n = 4$ experiments). Two-sided Student's unpaired-samples *t*-test; *$p = 0.046$ (DMSO versus dose 3 MLN8237), *$p = 0.012$ (DMSO versus dose 1 BI2536), **$p = 0.007$ (DMSO versus dose 2 MLN8237), **$p = 0.006$ (DMSO versus dose 1 D4476), **$p = 0.004$ (DMSO versus dose 2 D4476), and ****$p < 0.0001$. **c**, Quantification of daughter cell-size ratio in HeLa cells exposed to vehicle or inhibitors as depicted in panel **b**. The cell size was normalized with inhibitors of AURKA and AURKB (AZD1152) (mean ± s.d; $n = 4$ experiments). Two-sided Student's unpaired-samples *t*-test; *$p = 0.015$, **$p = 0.003$, and ****$p < 0.0001$.

Supplementary Figure 22

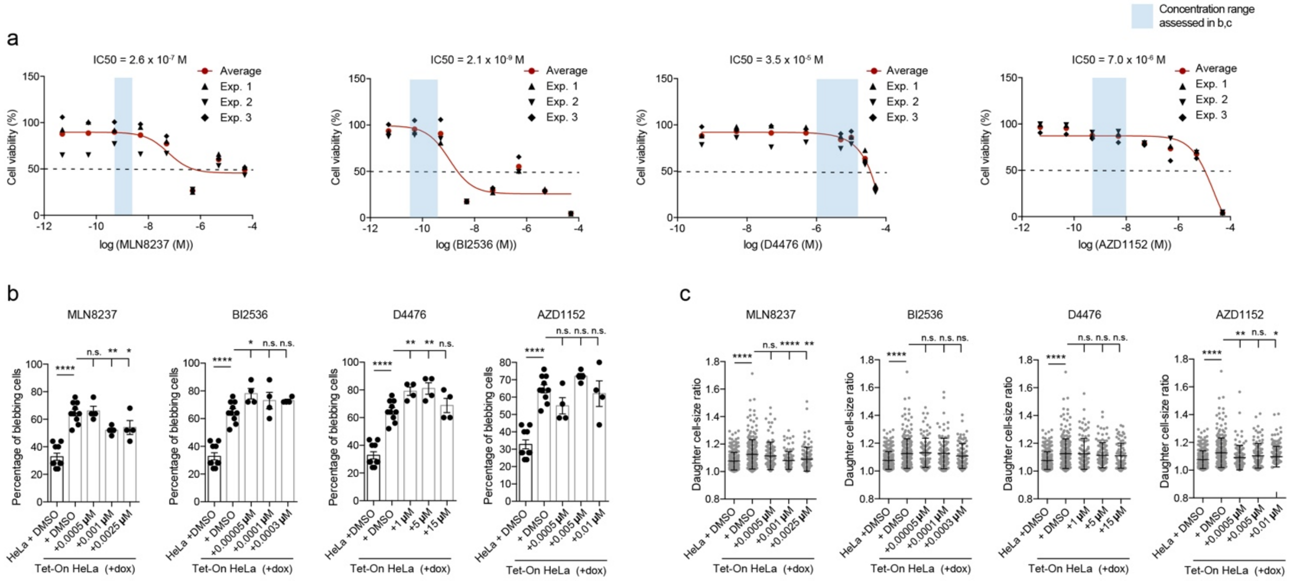

Supplementary Figure 23. HMMR overexpression in premalignant mammary tissue of *Blg-Cre*;*HMMR*$^{Tg/Tg}$;*Brca1*$^{f/f}$ mice. Left panel, cartoon representation (edited from Shuryak *et al.*, *PLoS One* 2013 Dec 20;8(12):e85795; doi:10.1371/journal.pone.0085795; distributed under Creative Commons Attribution License) of premalignant tissue taken from the mammary glands contralateral to the incident tumor. Middle panel, immunofluorescence analysis and quantitation of human HMMR in mouse mammary tissue. Scale bar = 20 μm. Right panel, tissue from six mice were examined per genotype and the mean value for each mouse is plotted (arbitrary units (arb. units) per μm^2; mean ± s.e.m.; frames n = 3 per tissue; each frame > 100 cells; n = 3 experiments). Two-tailed Student's t-test; *p = 0.024.

Supplementary Figure 23

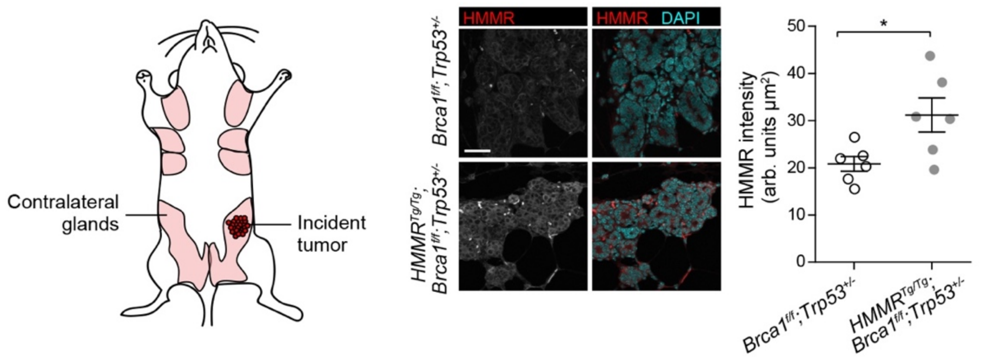

Supplementary Figure 24. Gene expression changes by HMMR overexpression in premalignant mammary tissue of *Blg-Cre*;*HMMR*$^{Tg/Tg}$;*Brca1*$^{f/f}$ mice. **a**, Overexpressed genes-biological processes ($p < 10^{-6}$) between the *Blg-Cre*;*HMMR*$^{Tg/Tg}$;*Brca1*$^{f/f}$;*Trp53*$^{+/-}$ relative to *Blg-Cre*;*Brca1*$^{f/f}$;*Trp53*$^{+/-}$ premalignant mammary tissue. **b**, Underexpressed genes-biological processes ($p < 10^{-6}$) between the *Blg-Cre*;*HMMR*$^{Tg/Tg}$;*Brca1*$^{f/f}$;*Trp53*$^{+/-}$ relative to *Blg-Cre*;*Brca1*$^{f/f}$;*Trp53*$^{+/-}$ premalignant mammary tissue.

Supplementary Figure 24

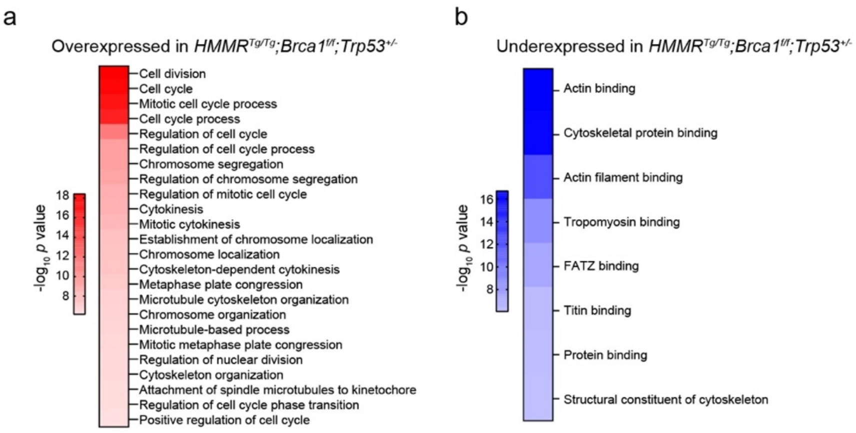

Supplementary Figure 25. Evaluation of mitotic and cycling cells in premalignant mammary tissue. **a**, Immunofluorescence analysis and quantitation of mitotic mammary epithelial cells as determined by TUBB staining. Premalignant mammary tissue from six mice were examined per genotype and the mean value for each mouse is plotted (mean ± s.e.m.; frames n = 3 per tissue; each frame > 100 cells; n = 3 experiments). Two-tailed Student's unpaired-samples t-test; $^{**}p$ = 0.008. **b**, Immunofluorescence analysis and quantitation of cyclin B1 (CCNB1). The tissue from six mice were examined per genotype and the mean value for each mouse is plotted (mean ± s.e.m.; 3 frames per tissue; > 100 cells per frame; n = 3 experiments). Two-tailed Student's unpaired-samples t-test; n.s. Scale bar = 10 μm.

Supplementary Figure 25

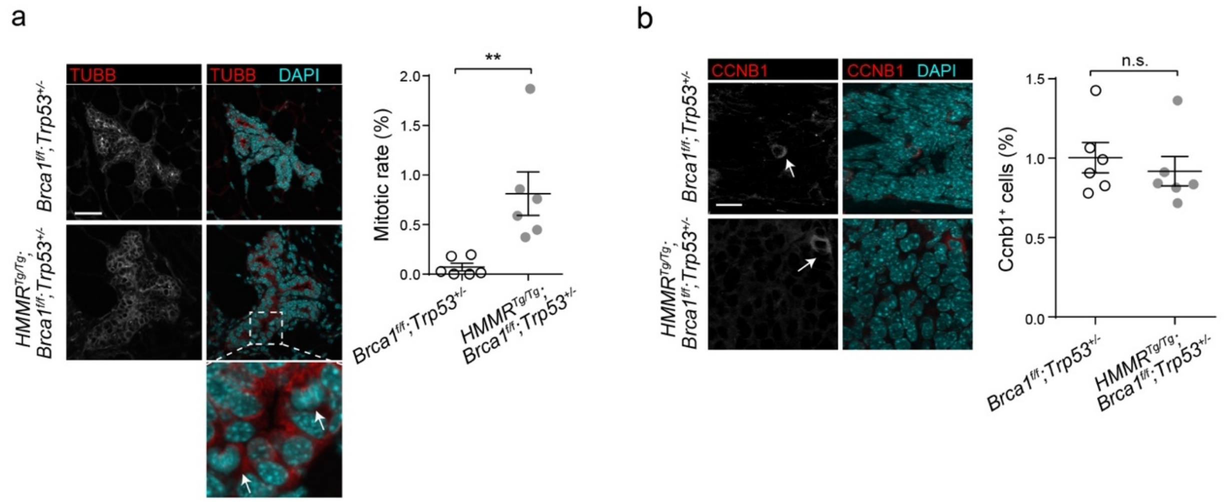

Supplementary Figure 26. HMMR-associated perturbations in premalignant progression of the *Blg-Cre*;*Brca1$^{f/f}$*;*Trp53$^{+/-}$* mammary tumorigenesis model. **a**, Violin plots displaying sample medians and outliers of *Hmmr* expression level (mouse endogenous) in single cells across premalignant stages (1 to 4; stage 5, tumor) in *Blg-Cre;Brca1$^{f/f}$;p53$^{+/-}$* mammary tissue (publicly available data, see Data availability section; mice $n = 3$ per time point; premalignant stages $n = 15$; tumors $n = 2$; approximately 100.000 cells total). The inset shows *Hmmr* expression across stages 1-4 considering only luminal progenitors (plot shows linked sample medians (red dots); stage 5 is not included as only contained 16 such cells). One-way ANOVA; *$p = 0.019$ and ****$p < 0.0001$. **b**, Violin plots of the expression level of defined gene sets (inset) across premalignant and tumor stages, using all single cell RNA-seq data. The three depicted pathways showed significant differences; Kruskal−Wallis test; ****$p < 0.0001$. "TNF signaling via NF-kB" and "KEGG cytosolic DNA sensing" increased, while "Reactome tight junction interactions" decreased through stages. In the box plots inside violin plots the horizontal lines represent the sample medians, the boxes extend from first to third quartile, the whiskers indicate values at 1.5 times the interquartile range, and there are shown outliers. The horizontal lines connect sample medians. **c**, Violin plots (format as panel **b**) of the expression level of defined gene sets (inset) across premalignant and tumor stages, using only RNA-seq data from luminal progenitors. Kruskal−Wallis test; ****$p < 0.0001$. "KEGG cytosolic DNA sensing" increased, while "Reactome tight junction interactions" decreased through stages.

Supplementary Figure 26

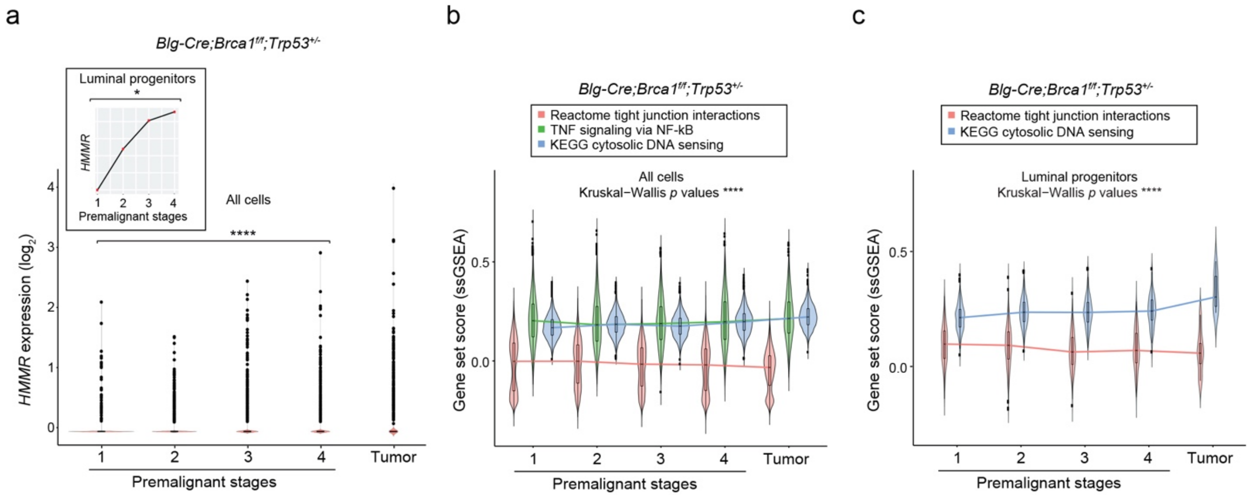

Supplementary Figure 27. HMMR overexpression causes immune-related gene expression changes in MECs, and cGAS inhibition causes the opposite for target genes. a, Plot of differentially expressed genes between *Blg-Cre*;*HMMR*$^{Tg/Tg}$;*Brca1*$^{f/f}$;*Trp53*$^{+/-}$ and *Blg-Cre*;*Brca1*$^{f/f}$;*Trp53*$^{+/-}$ MECs transduced with EGFP-Cre and examined by TaqMan Array Mouse Immune Response. Immune-related genes introduced are indicated, being overexpressed in *HMMR*$^{Tg/Tg}$ MECs. **b**, Analysis of downregulation of defined gene targets (panel **a**) in MECs exposed to cGAS inhibition at the published IC50 concentration (0.7 µM) or twice the IC50 concentration (1.4 µM). Significant downregulation of four of the tested genes was observed at the higher dose (mean ± s.d; n = 2 experiments; n = 2 wells per experiment). Two-sided Student's unpaired-samples t-test; *p = 0.018 (*Il1a*), **p = 0.007 (*Csf1*), **p = 0.006 (*Nfkb2*), and ****p < 0.0001 (*Nfkb1*).

Supplementary Figure 27

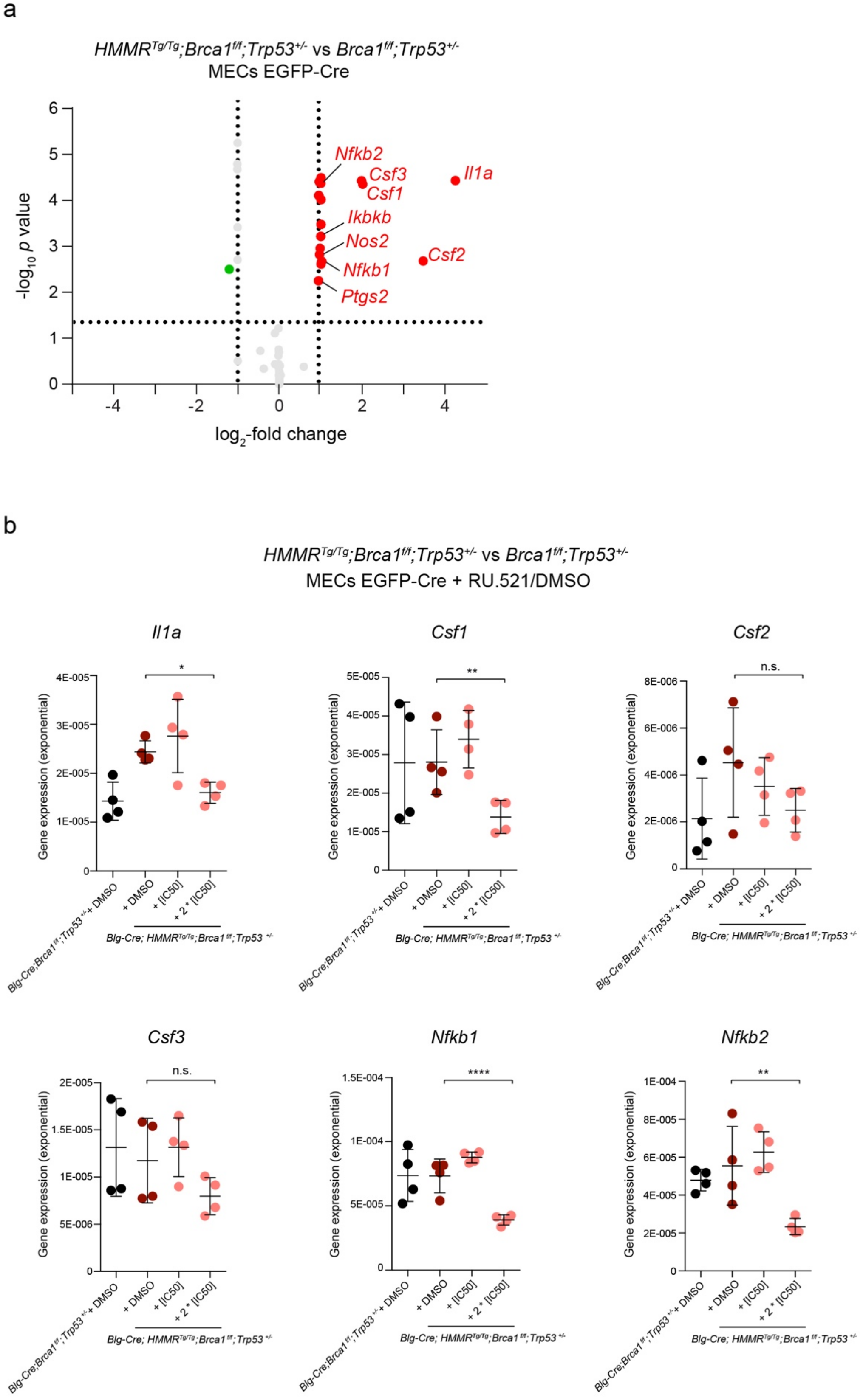

Supplementary Table 1a. GSEA gene sets overexpressed (nominal *p* < 0.001) in HMMRTg/Tg;Brca1f/f;Trp53+/- relative to Brca1f/f;Trp53+/- tumors.

Gene set	Size	ES	NES	*p*	FDR
TIAN_TNF_SIGNALING_VIA_NFKB	24	0.81	2.07	0.00E+00	5.62E-01
WILLIAMS_ESR2_TARGETS_UP	27	0.78	2.02	0.00E+00	4.30E-01
REACTOME_INTERLEUKIN_10_SIGNALING	42	0.7	2	0.00E+00	4.79E-01
CROMER_TUMORIGENESIS_UP	48	0.67	1.98	0.00E+00	5.08E-01
LIAN_LIPA_TARGETS_6M	66	0.64	1.98	0.00E+00	4.46E-01
HINATA_NFKB_TARGETS_KERATINOCYTE_UP	77	0.63	1.97	0.00E+00	3.61E-01
REACTOME_ERCC6_CSB_AND_EHMT2_G9A_POSITIVELY_REGULATE_RRNA_EXPRESSION	56	0.63	1.91	0.00E+00	4.80E-01
ZHANG_RESPONSE_TO_IKK_INHIBITOR_AND_TNF_UP	197	0.54	1.9	0.00E+00	4.78E-01
OSWALD_HEMATOPOIETIC_STEM_CELL_IN_COLLAGEN_GEL_UP	206	0.53	1.89	0.00E+00	5.22E-01
SCHUETZ_BREAST_CANCER_DUCTAL_INVASIVE_UP	311	0.51	1.87	0.00E+00	4.62E-01
ALTEMEIER_RESPONSE_TO_LPS_WITH_MECHANICAL_VENTILATION	106	0.57	1.87	0.00E+00	3.98E-01
SANA_TNF_SIGNALING_UP	69	0.61	1.86	0.00E+00	3.89E-01
LINDSTEDT_DENDRITIC_CELL_MATURATION_A	57	0.62	1.85	0.00E+00	4.18E-01
LINDGREN_BLADDER_CANCER_CLUSTER_2B	345	0.5	1.84	0.00E+00	4.59E-01
REACTOME_RHO_GTPASES_ACTIVATE_PKNS	72	0.59	1.82	0.00E+00	4.81E-01
ANASTASSIOU_MULTICANCER_INVASIVENESS_SIGNATURE	63	0.59	1.81	0.00E+00	4.80E-01
PLASARI_TGFB1_TARGETS_10HR_UP	187	0.51	1.8	0.00E+00	4.85E-01
HOLLERN_EMT_BREAST_TUMOR_UP	130	0.53	1.79	0.00E+00	5.17E-01
WESTON_VEGFA_TARGETS	95	0.55	1.75	0.00E+00	5.83E-01
QI_PLASMACYTOMA_UP	241	0.48	1.75	0.00E+00	5.92E-01
VART_KSHV_INFECTION_ANGIOGENIC_MARKERS_UP	154	0.51	1.74	0.00E+00	5.74E-01
PHONG_TNF_RESPONSE_VIA_P38_PARTIAL	156	0.49	1.71	0.00E+00	5.75E-01
ZWANG_CLASS_3_TRANSIENTLY_INDUCED_BY_EGF	205	0.48	1.68	0.00E+00	5.79E-01
VERHAAK_GLIOBLASTOMA_MESENCHYMAL	194	0.47	1.66	0.00E+00	5.67E-01
NAGASHIMA_NRG1_SIGNALING_UP	162	0.47	1.65	0.00E+00	5.75E-01
PICCALUGA_ANGIOIMMUNOBLASTIC_LYMPHOMA_UP	190	0.47	1.64	0.00E+00	5.75E-01
REACTOME_ESR_MEDIATED_SIGNALING	198	0.47	1.64	0.00E+00	5.62E-01
CHARAFE_BREAST_CANCER_LUMINAL_VS_MESENCHYMAL_DN	437	0.42	1.61	0.00E+00	5.89E-01
REACTOME_EXTRACELLULAR_MATRIX_ORGANIZATION	286	0.44	1.6	0.00E+00	5.74E-01
PHONG_TNF_RESPONSE_NOT_VIA_P38	320	0.43	1.59	0.00E+00	5.58E-01
OISHI_CHOLANGIOMA_STEM_CELL_LIKE_DN	257	0.43	1.55	0.00E+00	5.73E-01
SMID_BREAST_CANCER_NORMAL_LIKE_UP	416	0.39	1.49	0.00E+00	5.66E-01

Supplementary Table 1b. GSEA gene sets underexpressed (nominal *p* < 0.05) in HMMRTg/Tg;Brca1f/f;Trp53+/- relative to Brca1f/f;Trp53+/- tumors.

Gene set	Size	ES	NES	*p*	FDR
REACTOME_FORMATION_OF_THE_CORNIFIED_ENVELOPE	79	-0.51	-1.64	2.00E-03	1.00E+00
NIKOLSKY_BREAST_CANCER_7P22_AMPLICON	36	-0.66	-1.89	4.00E-03	1.00E+00
KEGG_GLUTATHIONE_METABOLISM	45	-0.54	-1.65	4.00E-03	1.00E+00
REACTOME_KERATINIZATION	82	-0.49	-1.62	9.00E-03	1.00E+00
SENGUPTA_NASOPHARYNGEAL_CARCINOMA_DN	292	-0.36	-1.39	9.00E-03	1.00E+00
REACTOME_ABC_TRANSPORTERS_IN_LIPID_HOMEOSTASIS	17	-0.72	-1.77	1.00E-02	1.00E+00
CHIANG_LIVER_CANCER_SUBCLASS_PROLIFERATION_DN	142	-0.41	-1.47	1.10E-02	1.00E+00
REACTOME_TIGHT_JUNCTION_INTERACTIONS	26	-0.63	-1.69	1.60E-02	1.00E+00
LIM_MAMMARY_STEM_CELL_DN	390	-0.34	-1.33	1.60E-02	1.00E+00
REACTOME_BILE_ACID_AND_BILE_SALT_METABOLISM	34	-0.54	-1.52	2.30E-02	1.00E+00
LIM_MAMMARY_LUMINAL_PROGENITOR_UP	55	-0.48	-1.51	2.60E-02	1.00E+00
REACTOME_GLUTATHIONE_CONJUGATION	33	-0.55	-1.52	2.80E-02	1.00E+00
MIKKELSEN_MEF_HCP_WITH_H3_UNMETHYLATED	183	-0.37	-1.34	2.80E-02	1.00E+00
YUAN_ZNF143_PARTNERS	22	-0.59	-1.56	2.90E-02	1.00E+00
REACTOME_SYNTHESIS_OF_BILE_ACIDS_AND_BILE_SALTS	28	-0.57	-1.52	2.90E-02	1.00E+00
NIKOLSKY_BREAST_CANCER_17Q11_Q21_AMPLICON	106	-0.4	-1.36	3.30E-02	1.00E+00
EBAUER_TARGETS_OF_PAX3_FOXO1_FUSION_UP	181	-0.36	-1.32	4.20E-02	1.00E+00
KEGG_ABC_TRANSPORTERS	38	-0.51	-1.47	4.30E-02	1.00E+00
REACTOME_MISCELLANEOUS_TRANSPORT_AND_BINDING_EVENTS	25	-0.58	-1.52	4.40E-02	1.00E+00
YAMASHITA_LIVER_CANCER_STEM_CELL_DN	58	-0.46	-1.43	4.90E-02	1.00E+00

Supplementary Table 2. Primers and TaqMan™ probes used in this study.

Gene	Accession number	Forward 5'-3'	Reverse 5'-3'
Brca1	NM_009764.3	CCCTCAAGAAGCTGGAGATG	TGCCCTCAGAAAACTCACAA
Il10	NM_010548.2	CCCCTGTGAAAATAAGAGCAA	TGCAGTTGATGAAGATGTCAAA
Il6	NM_031168.2	TGATGGATGCTACCAAACTGG	TTCATGTACTCCAGGTAGCTATGG
Ppia	NM_008907.2	CAAATGCTGGACCAAACACAAACG	GTTCATGCCTTCTTTCACCTTCCC
Slug/Snai2	NM_011415.3	TGGTCAAGAAACATTTCAACGCC	GGTGAGGATCTCTGGTTTTGGTA
Sparc	NM_009242.5	TGTCCTGGTCACCTTGTACG	CAGGCGCTTCTCATTCTCAT
Trp53	NM_011640.3	CTCTCCCCCGCAAAAGAAAAA	CGGAACATCTCGAAGCGTTTA
Twist1	NM_011658.2	GGACAAGCTGAGCAAGATTCA	CGGAGAAGGCGTAGCTGAG
Vegfa	NM_001025250.3	TGTACCTCCACCATGCCAAGT	TGGTAGACATCCATGAACTTG
Vim	NM_011701.4	CGTCCACACGCACCTACAG	GGGGGATGAGGAATAGAGGCT
**TaqMan™ (Thermo Fisher Scientific)**			
Hs00234864	Catalog 4331182	Human *HMMR*	
Hs01063269	Catalog 4351372	Human *HMMR*	
Hs01063280	Catalog 4351372	Human *HMMR*	
Mm00469183	Catalog 4448489	Mouse *Hmmr*	
Mm99999915	Catalog 4331182	Mouse *Gapdh*	

Supplementary Table 3. Antibodies used in this study.

Antibody	Manufacturer	Reference	Species	Application*	Dilution	Retrieval
Anti-ARPC2	Santa Cruz Biotechnologies	sc-515754	Mouse	IF	1:200	
Anti-phospho-Aurora A (Thr288)	Cell Signaling	3079	Rabbit	IF	1:1000	
Anti-BRCA1	EMD Millipore	OP92	Mouse	IF	1:20	
Anti-β-Tubulin (9F3) (Alexa Fluor®647 conjugate)	Cell Signaling	3624	Rabbit	IF	1:300	
Anti-CCNB1	Cell Signaling	4138	Rabbit	IF	1:500	
Anti-CD31	Abcam	ab28364	Rabbit	IHC	1:50	Citrate pH6
Anti-mouse CD45 (Alexa Fluor®488 conjugate)	Biolegend	103121	Rat	IF	1:100	
Anti-CD45R	Abcam	ab64100	Rat	IHC	1:100	Citrate pH6
Anti-CD68 (Alexa Fluor®594 conjugate)	Biolegend	137020	Rat	IF	1:100	
Anti-CD106 (Alexa Fluor®647 conjugate)	BD	561612	Rat	IF	1:100	
Anti-cGAS (D3O8O)	Cell Signaling	31659	Rabbit	IF	1:500	
Anti-cGAS (D1D3G)	Cell Signaling	15102	Rabbit	IF	1:500	
Anti-CLDN1/3 (MH25)	Invitrogen	71-7800	Rabbit	IHC	1:20	Citrate pH6
Anti-ER□	Invitrogen	MA5-13304	Mouse	IHC	1:40	Citrate pH6
Anti-F4/80 (Alexa Fluor®488 conjugate)	Biolegend	123120	Rat	IF	1:100	
Anti-GAPDH	Proteintech/Cedarlane Labs	60004-1-Ig	Mouse	WB	1:50000	
Anti-HMMR	Abcam	ab124729	Rabbit	IF	1:1000	
Anti-HMMR	Abcam	ab124729	Rabbit	WB	1:1000	
Anti-HMMR	Abcam	ab108339	Rabbit	IF	1:1000	
Anti-K8	DSHB	TROMA-I	Rat	IHC	1:100	Citrate pH6
Anti-K14	Biolegend	905301	Rabbit	IHC	1:200	Citrate pH6
Anti-LMNB1	Abcam	Ab16048	Rabbit	IF		
Anti-NFkB p65	Abcam	Ab16502	Rabbit	IF	1:500	
Anti-NFkB p52	EMD Millipore	06-413	Rabbit	IF	1:500	
Anti-NMIIa/MYH9 (2B3)	Abcam	ab55456	Mouse	IF	1:100	
Anti-NMIIb/MYH10 (3H2)	Abcam	ab684	Mouse	IF	1:50	
Anti-PCNT	Biolegend	PRB-923701	Rabbit	IF	1:500	
Anti-p34-ARC antibody (F5)	Santa Cruz Biotechnologies	sc-515754	Mouse	IF	1:200	
Anti-Vimentin/VIM	Cell Signaling	5741	Rabbit	IF	1:500	
Anti-Vimentin/VIM	Abcam	ab92547	Rabbit	IHC	1:200	Citrate pH6
Anti-ZO1	Invitrogen	40-2200	Mouse	IF	1:500	

*IF: immunofluorescence; IHC, immunohistochemistry; WB, western blotting.

Original gel images Supplementary Figure 4a (included in Source data)

HMMR$^{Tg/+}$

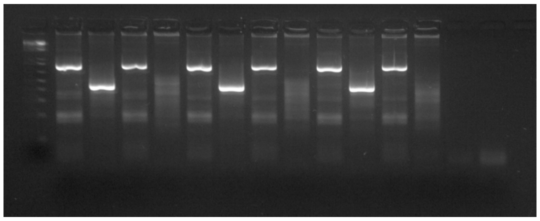

HMMR$^{Tg/Tg}$

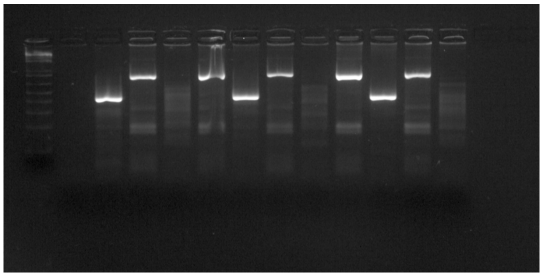

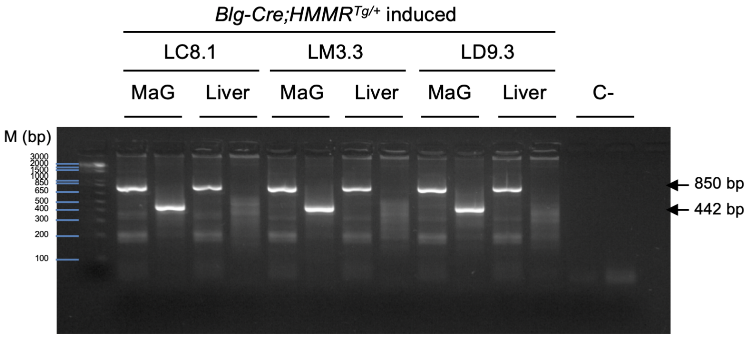

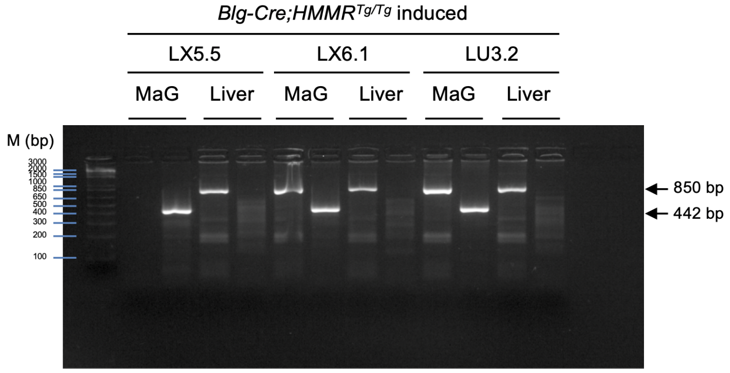

Uncropped images Supplementary Figure 4b (included in Source data)

HMMR

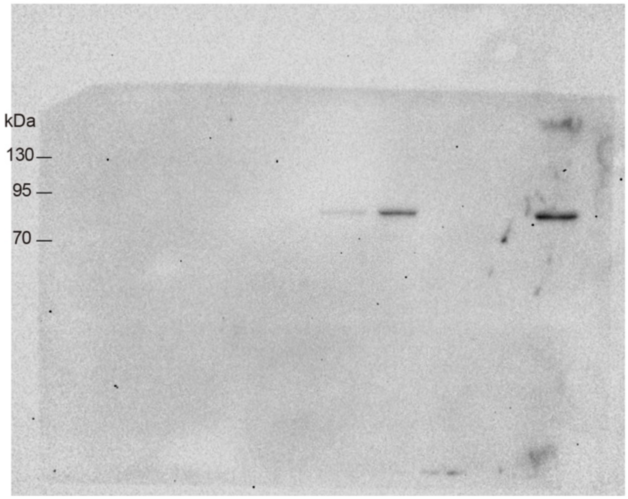

TUBA

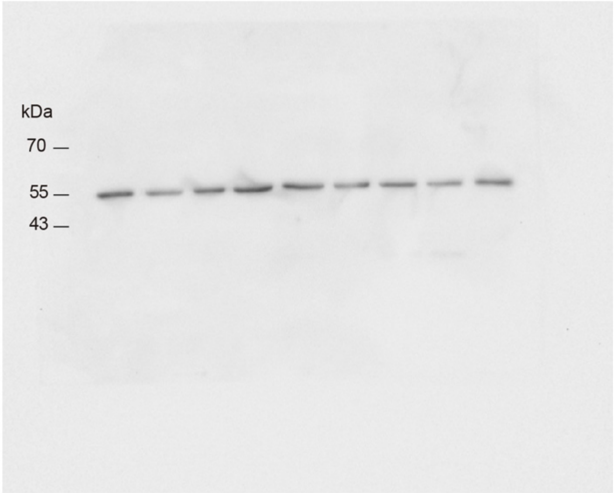

Original gel images Supplementary Figure 6 (included in Source data)

HMMR

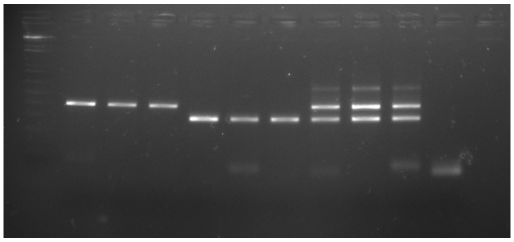

Brca1

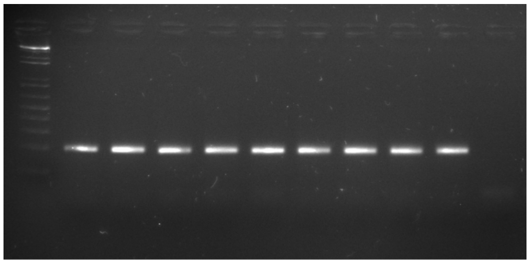

Trp53

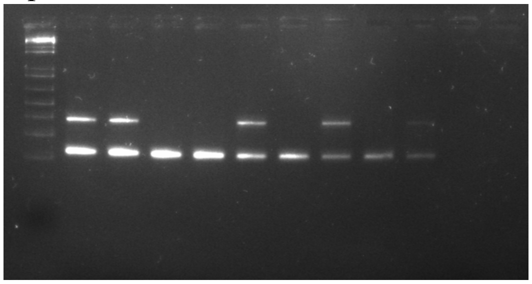

Cre

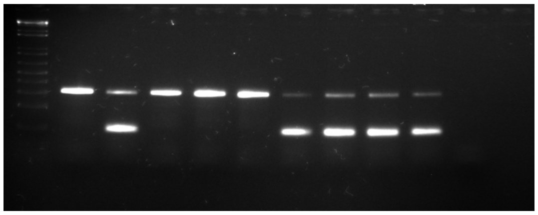

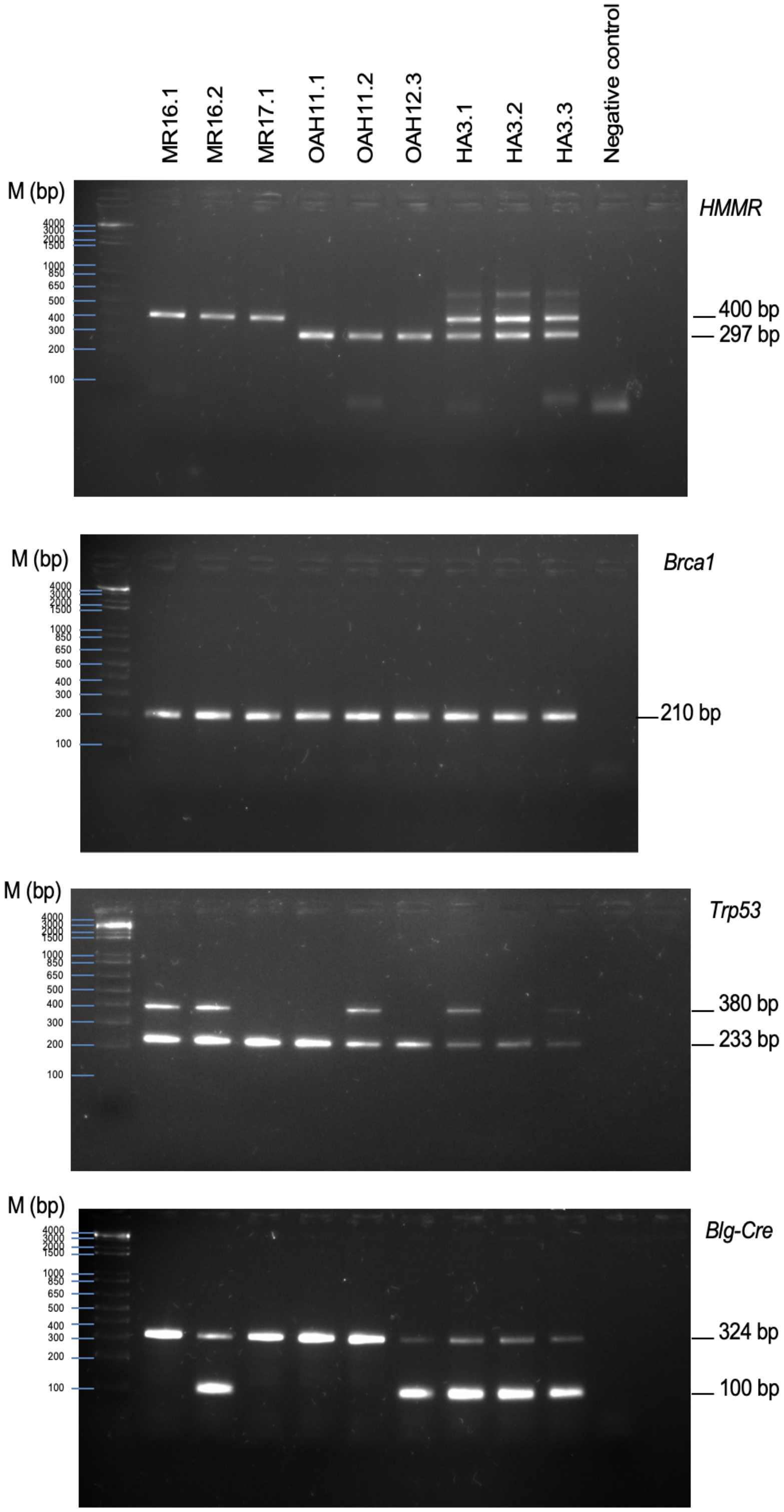

Original gel images Supplementary Figure 7 (included in Source data)

HMMR

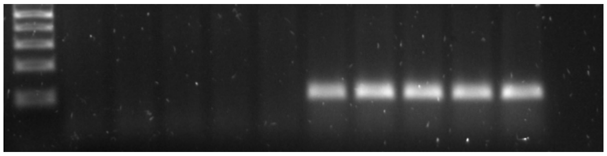

Ppia

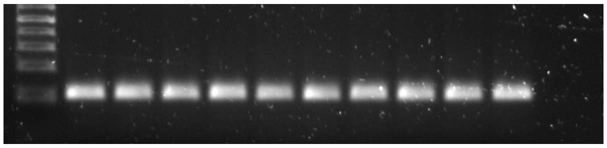

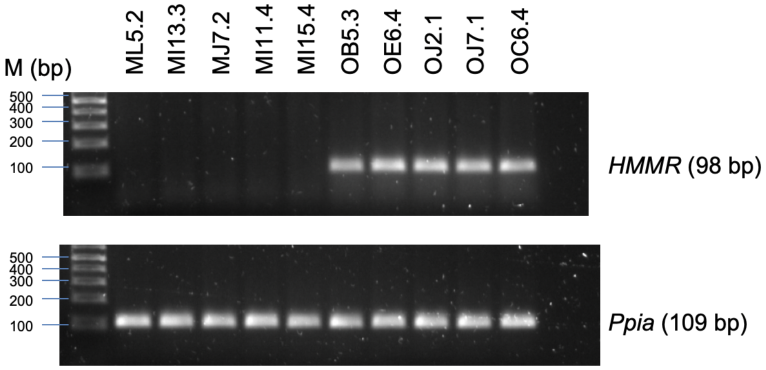

Uncropped images Supplementary Figure 15a (also in Source data)

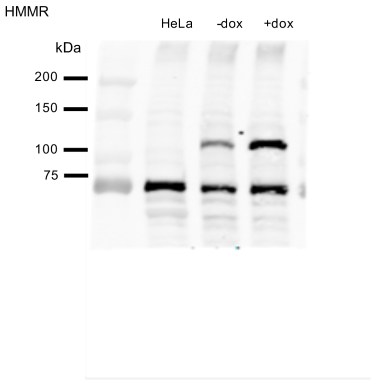

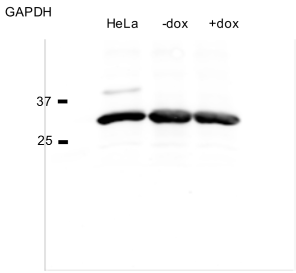

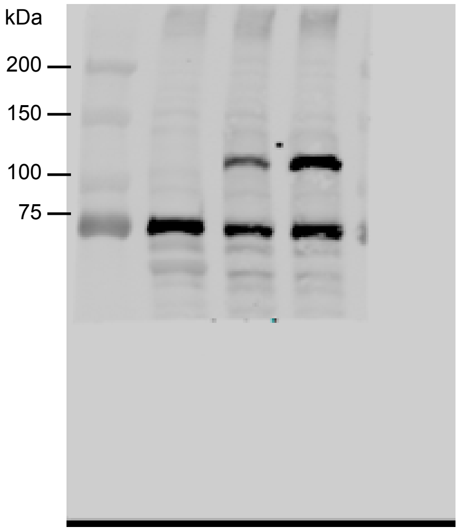

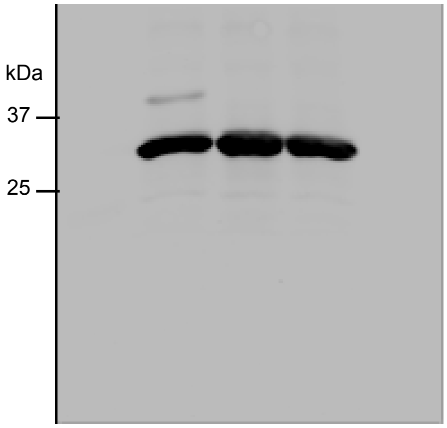

Uncropped images Supplementary Figure 20 (included in Source data)

Source data Fig S20

HMMR IP to examine co-immunoprecipitation with ARP2/3

1) Synchronization: Nocodazole for 16 hours + MG132 for 2 hours

Lane	Sample
1	WCL
2	WCL cleared
3	Post-IP IgG
4	IP IgG
5	Post-IP HMMR
6	IP HMMR

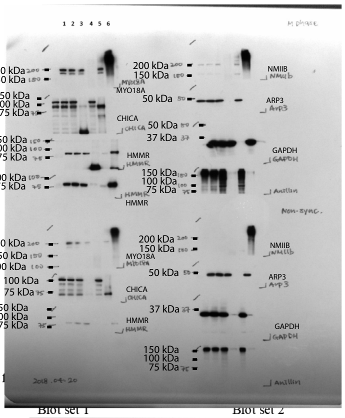

* IB as labeled, ladder size is labeled on the left side. Ladder marked on the right hand side of each blot (kDa).

Short exposure time

Long exposure time
M-phase

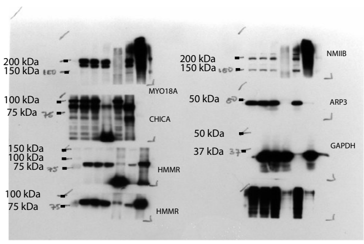

2) Synchronization: STLC f

Lane	Sample
1	WCL
2	WCL cleared
3	Post-IP IgG
4	Post-IP HMMR
5	IP IgG
6	IP HMMR

Blot set 1 Blot set 2

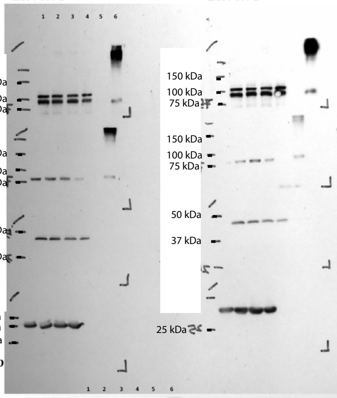

Short exposure time

IB: CHICA

IB: HMMR

IB: ARP3

IB: GAPDH

Blot set 1 Blot set 2

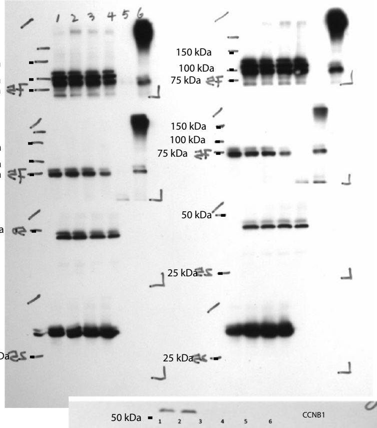

Long exposure time

IB: CHICA

IB: HMMR

IB: ARP3

IB: GAPDH

3) Synchronization: Cdk1 inhib

size is labeled on the left side.

Lane	Sample
1	WCL
2	WCL cleared
3	Post-IP IgG
4	Post-IP HMM
5	IP IgG
6	IP HMMR

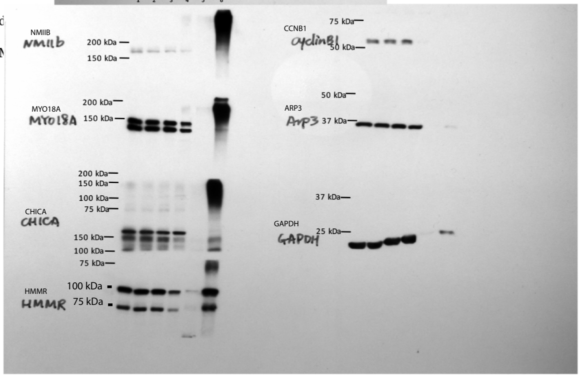

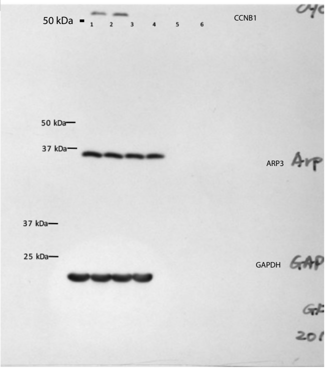

*Blot run in parallel

IB: ARP3

IB: GAPDH

CIMBA Acknowledgements

<u>Funding</u>

The CIMBA data management and data analysis were supported by Cancer Research – UK grants PPRPGM-Nov20\100002 and C12292/A20861 and the Gray Foundation. GCT and ABS are NHMRC Research Fellows. iCOGS: the European Community's Seventh Framework Programme under grant agreement n° 223175 (HEALTH-F2-2009-223175) (COGS), Cancer Research UK (C1287/A10118, C1287/A 10710, C12292/A11174, C1281/A12014, C5047/A8384, C5047/A15007, C5047/A10692, C8197/A16565), the National Institutes of Health (CA128978) and Post-Cancer GWAS initiative (1U19 CA148537, 1U19 CA148065 and 1U19 CA148112 - the GAME-ON initiative), the Department of Defence (W81XWH-10-1-0341), the Canadian Institutes of Health Research (CIHR) for the CIHR Team in Familial Risks of Breast Cancer (CRN-87521), and the Ministry of Economic Development, Innovation and Export Trade (PSR-SIIRI-701), Komen Foundation for the Cure, the Breast Cancer Research Foundation, and the Ovarian Cancer Research Fund. OncoArray: the PERSPECTIVE and PERSPECTIVE I&I projects funded by the Government of Canada through Genome Canada and the Canadian Institutes of Health Research, the 'Ministère de l'Économie, de la Science et de l'Innovation du Québec' through Genome Québec, and the Quebec Breast Cancer Foundation; the NCI Genetic Associations and Mechanisms in Oncology (GAME-ON) initiative and Discovery, Biology and Risk of Inherited Variants in Breast Cancer (DRIVE) project (NIH Grants U19 CA148065 and X01HG007492); and Cancer Research UK (C1287/A10118 and C1287/A16563). BCFR: UM1 CA164920 from the National Cancer Institute. The content of this manuscript does not necessarily reflect the views or policies of the National Cancer Institute or any of the collaborating centers in the Breast Cancer Family Registry (BCFR), nor does mention of trade names, commercial products, or organizations imply endorsement by the US Government or the BCFR. BFBOCC: Lithuania (BFBOCC-LT): Research Council of Lithuania grant SEN-18/2015. BIDMC: Breast Cancer Research Foundation. BMBSA: Cancer Association of South Africa (PI Elizabeth J. van Rensburg). CNIO: CNIO study is partially funded by FIS PI19/00640 supported by FEDER funds and the Spanish Network on Rare Diseases (CIBERER). CCGCRN: Research reported in this publication was supported by the Breast Cancer Research Foundation (project 20-172), National Cancer Institute of the National Institutes of Health under grant number R25CA112486, and RC4CA153828 (PI: J. Weitzel) from the National Cancer Institute and the Office of the Director, National Institutes of Health. The content is solely the responsibility of the authors and does not necessarily represent the official views of the National Institutes of Health. CONSIT TEAM: Associazione Italiana Ricerca sul Cancro (AIRC; IG2015 no.16732) to P. Peterlongo**.** DEMOKRITOS: European Union (European Social Fund – ESF) and Greek national funds through the Operational Program "Education and Lifelong Learning" of the National Strategic Reference Framework (NSRF) - Research Funding Program of the General Secretariat for Research & Technology: SYN11_10_19 NBCA. Investing in knowledge society through the European Social Fund. DFKZ: German Cancer Research Center. EMBRACE: Cancer Research UK Grants C1287/A23382 and C1287/A26886. D. Gareth Evans and Fiona Lalloo are supported by an NIHR grant to the Biomedical Research Centre, Manchester. The Investigators at The Institute of Cancer Research and The Royal Marsden NHS Foundation Trust are supported by an NIHR grant to the Biomedical Research Centre at The Institute of Cancer Research and The Royal Marsden NHS Foundation Trust. Ros Eeles and Elizabeth Bancroft are supported by Cancer Research UK Grant C5047/A8385. Ros Eeles is also supported by NIHR support to the Biomedical Research Centre at The Institute of Cancer Research and The Royal Marsden NHS Foundation Trust. FCCC The University of Kansas Cancer Center (P30 CA168524), the Kansas Institute for Precision Medicine

(P20GM130423) and the Kansas Bioscience Authority Eminent Scholar Program. A.K.G. was funded by R01CA140323, R01CA214545, R01CA260132, 5U10CA180888, and by the Chancellors Distinguished Chair in Biomedical Sciences Professorship. FPGMX: A.Vega is supported by the Spanish Health Research Foundation, Instituto de Salud Carlos III (ISCIII), partially supported by FEDER funds through Research Activity Intensification Program (contract grant numbers: INT15/00070, INT16/00154, INT17/00133), and through Centro de Investigación Biomédica en Red de Enferemdades Raras CIBERER (ACCI 2016: ER17P1AC7112/2018); Autonomous Government of Galicia (Consolidation and structuring program: IN607B), and by the Fundación Mutua Madrileña. GC-HBOC: German Cancer Aid (grant no 110837 and 113049, Rita K. Schmutzler) and the European Regional Development Fund and Free State of Saxony, Germany (LIFE - Leipzig Research Centre for Civilization Diseases, project numbers 713-241202, 713-241202, 14505/2470, 14575/2470). GEMO: Ligue Nationale Contre le Cancer; the Association "Le cancer du sein, parlons-en!" Award, the Canadian Institutes of Health Research for the "CIHR Team in Familial Risks of Breast Cancer" program, the Fondation ARC pour la recherche sur le cancer (grant PJA 20151203365) and the French National Institute of Cancer (INCa grants AOR 01 082, 2001-2003, 2013-1-BCB-01-ICH-1 and SHS-E-SP 18-015). GEORGETOWN: the Non-Therapeutic Subject Registry Shared Resource at Georgetown University (NIH/NCI grant P30-CA051008), the Fisher Center for Hereditary Cancer and Clinical Genomics Research, and Swing Fore the Cure. G-FAST: Bruce Poppe is a senior clinical investigator of FWO. Mattias Van Heetvelde obtained funding from IWT. HCSC: Spanish Ministry of Health PI15/00059, PI16/01292, and CB-161200301 CIBERONC from ISCIII (Spain), partially supported by European Regional Development FEDER funds. HEBCS: Helsinki University Hospital Research Fund, the Finnish Cancer Society and the Sigrid Juselius Foundation. HEBON: the Dutch Cancer Society grants NKI1998-1854, NKI2004-3088, NKI2007-3756, the Netherlands Organization of Scientific Research grant NWO 91109024, the Pink Ribbon grants 110005 and 2014-187.WO76, the BBMRI grant NWO 184.021.007/CP46 and the Transcan grant JTC 2012 Cancer 12-054. HEBON thanks the registration teams of Dutch Cancer Registry (IKNL; S. Siesling, J. Verloop) and the Dutch Pathology database (PALGA; L. Overbeek) for part of the data collection. HRBCP: Hong Kong Sanatorium and Hospital, Dr Ellen Li Charitable Foundation, The Kerry Group Kuok Foundation, National Institute of Health1R 03CA130065, and North California Cancer Center. HUNBOCS: Hungarian Research Grants KTIA-OTKA CK-80745 and NKFI_OTKA K-112228. ICO: The authors would like to particularly acknowledge the support of the Asociación Española Contra el Cáncer (AECC), the Instituto de Salud Carlos III (organismo adscrito al Ministerio de Economía y Competitividad) and "Fondo Europeo de Desarrollo Regional (FEDER), una manera de hacer Europa" (PI10/01422, PI13/00285, PIE13/00022, PI15/00854, PI16/00563 and CIBERONC) and the Institut Català de la Salut and Autonomous Government of Catalonia (2009SGR290, 2014SGR338 and PERIS Project MedPerCan). IHCC: PBZ_KBN_122/P05/2004. ILUH: Icelandic Association "Walking for Breast Cancer Research" and by the Landspitali University Hospital Research Fund. INHERIT: Canadian Institutes of Health Research for the "CIHR Team in Familial Risks of Breast Cancer" program – grant # CRN-87521 and the Ministry of Economic Development, Innovation and Export Trade – grant # PSR-SIIRI-701. IOVHBOCS: Ministero della Salute and "5x1000" Istituto Oncologico Veneto grant. IPOBCS: Liga Portuguesa Contra o Cancro. kConFab: The National Breast Cancer Foundation, and previously by the National Health and Medical Research Council (NHMRC), the Queensland Cancer Fund, the Cancer Councils of New South Wales, Victoria, Tasmania and South Australia, and the Cancer Foundation of Western Australia. KOHBRA: the Korea Health Technology R&D Project through the Korea Health Industry Development Institute (KHIDI), and the National R&D Program for Cancer Control, Ministry of Health & Welfare, Republic of Korea (HI16C1127; 1020350; 1420190).

KUMC: NIGMS P20 GM130423 (to AKG). MAYO: NIH grants CA116167, CA192393 and CA176785, an NCI Specialized Program of Research Excellence (SPORE) in Breast Cancer (CA116201),and a grant from the Breast Cancer Research Foundation. MCGILL: Jewish General Hospital Weekend to End Breast Cancer, Quebec Ministry of Economic Development, Innovation and Export Trade. Marc Tischkowitz is supported by the funded by the European Union Seventh Framework Program (2007Y2013)/European Research Council (Grant No. 310018). MODSQUAD: MH CZ - DRO (MMCI, 00209805), MEYS - NPS I - LO1413 to LF, and by Charles University in Prague project UNCE204024 (MZ). MSKCC: the Breast Cancer Research Foundation, the Robert and Kate Niehaus Clinical Cancer Genetics Initiative, the Andrew Sabin Research Fund and a Cancer Center Support Grant/Core Grant (P30 CA008748). NAROD: 1R01 CA149429-01. NCI: the Intramural Research Program of the US National Cancer Institute, NIH, and by support services contracts NO2-CP-11019-50, N02-CP-21013-63 and N02-CP-65504 with Westat, Inc, Rockville, MD. NICCC: Clalit Health Services in Israel, the Israel Cancer Association and the Breast Cancer Research Foundation (BCRF), NY. NNPIO: the Russian Foundation for Basic Research (grants 17-54-12007, 17-00-00171 and 18-515-12007). NRG Oncology: U10 CA180868, NRG SDMC grant U10 CA180822, NRG Administrative Office and the NRG Tissue Bank (CA 27469), the NRG Statistical and Data Center (CA 37517) and the Intramural Research Program, NCI. OSUCCG: Ohio State University Comprehensive Cancer Center. PBCS: Italian Association of Cancer Research (AIRC) [IG 2013 N.14477] and Tuscany Institute for Tumors (ITT) grant 2014-2015-2016. SEABASS: Ministry of Science, Technology and Innovation, Ministry of Higher Education (UM.C/HlR/MOHE/06) and Cancer Research Initiatives Foundation. SMC: the Israeli Cancer Association. SWE-BRCA: the Swedish Cancer Society. UCHICAGO: NCI Specialized Program of Research Excellence (SPORE) in Breast Cancer (CA125183), R01 CA142996, 1U01CA161032 and by the Ralph and Marion Falk Medical Research Trust, the Entertainment Industry Fund National Women's Cancer Research Alliance and the Breast Cancer research Foundation. OIO is an ACS Clinical Research Professor. UCLA: Jonsson Comprehensive Cancer Center Foundation; Breast Cancer Research Foundation. UCSF: UCSF Cancer Risk Program and Helen Diller Family Comprehensive Cancer Center. UKFOCR: Cancer Researc h UK. UPENN: Breast Cancer Research Foundation; Susan G. Komen Foundation for the cure, Basser Research Center for BRCA. UPITT/MWH: Hackers for Hope Pittsburgh. VFCTG: Victorian Cancer Agency, Cancer Australia, National Breast Cancer Foundation. WCP: Dr Karlan is funded by the American Cancer Society Early Detection Professorship (SIOP-06-258-01-COUN) and the National Center for Advancing Translational Sciences (NCATS), Grant UL1TR000124.

Acknowledgements

All the families and clinicians who contribute to the studies; Catherine M. Phelan for her contribution to CIMBA until she passed away on 22 September 2017; Sue Healey, in particular taking on the task of mutation classification with the late Olga Sinilnikova; Maggie Angelakos, Judi Maskiell, Gillian Dite, Helen Tsimiklis; members and participants in the New York site of the Breast Cancer Family Registry; members and participants in the Ontario Familial Breast Cancer Registry; Vilius Rudaitis and Laimonas Griškevičius; Drs Janis Eglitis, Anna Krilova and Aivars Stengrevics; Yuan Chun Ding and Linda Steele for their work in participant enrollment and biospecimen and data management; Bent Ejlertsen and Anne-Marie Gerdes for the recruitment and genetic counseling of participants; Alicia Barroso, Rosario Alonso and Guillermo Pita; all the individuals and the researchers who took part in CONSIT TEAM (Consorzio Italiano Tumori Ereditari Alla Mammella), in particular: Dario Zimbalatti, Daniela Zaffaroni, Laura Ottini, Giuseppe Giannini, Laura Papi, Gabriele Lorenzo Capone, Liliana Varesco, Viviana Gismondi, Maria

Grazia Tibiletti, Daniela Furlan, Antonella Savarese, Aline Martayan, Stefania Tommasi, Brunella Pilato and the personnel of the Cogentech Cancer Genetic Test Laboratory, Milan, Italy. Ms. JoEllen Weaver and Dr. Betsy Bove; FPGMX: members of the Cancer Genetics group (IDIS): Ana Blanco, Miguel Aguado, Uxía Esperón and Belinda Rodríguez; IFE - Leipzig Research Centre for Civilization Diseases (Markus Loeffler, Joachim Thiery, Matthias Nüchter, Ronny Baber); We thank all participants, clinicians, family doctors, researchers, and technicians for their contributions and commitment to the DKFZ study and the collaborating groups in Lahore, Pakistan (Muhammad U. Rashid, Noor Muhammad, Sidra Gull, Seerat Bajwa, Faiz Ali Khan, Humaira Naeemi, Saima Faisal, Asif Loya, Mohammed Aasim Yusuf) and Bogota, Colombia (Diana Torres, Ignacio Briceno, Fabian Gil). Genetic Modifiers of Cancer Risk in BRCA1/2 Mutation Carriers (GEMO) study is a study from the National Cancer Genetics Network UNICANCER Genetic Group, France. We wish to pay a tribute to Olga M. Sinilnikova, who with Dominique Stoppa-Lyonnet initiated and coordinated GEMO until she sadly passed away on the 30th June 2014. The team in Lyon (Olga Sinilnikova, Mélanie Léoné, Laure Barjhoux, Carole Verny-Pierre, Sylvie Mazoyer, Francesca Damiola, Valérie Sornin) managed the GEMO samples until the biological resource centre was transferred to Paris in December 2015 (Noura Mebirouk, Fabienne Lesueur, Dominique Stoppa-Lyonnet). We want to thank all the GEMO collaborating groups for their contribution to this study: Coordinating Centre, Service de Génétique, Institut Curie, Paris, France: Muriel Belotti, Ophélie Bertrand, Anne-Marie Birot, Bruno Buecher, Sandrine Caputo, Chrystelle Colas, Anaïs Dupré, Emmanuelle Fourme, Marion Gauthier-Villars, Lisa Golmard, Marine Le Mentec, Virginie Moncoutier, Antoine de Pauw, Claire Saule, Dominique Stoppa-Lyonnet, and Inserm U900, Institut Curie, Paris, France: Fabienne Lesueur, Noura Mebirouk.Contributing Centres : Unité Mixte de Génétique Constitutionnelle des Cancers Fréquents, Hospices Civils de Lyon - Centre Léon Bérard, Lyon, France: Nadia Boutry-Kryza, Alain Calender, Sophie Giraud, Mélanie Léone. Institut Gustave Roussy, Villejuif, France: Brigitte Bressac-de-Paillerets, Olivier Caron, Marine Guillaud-Bataille. Centre Jean Perrin, Clermont–Ferrand, France: Yves-Jean Bignon, Nancy Uhrhammer. Centre Léon Bérard, Lyon, France: Valérie Bonadona, Christine Lasset. Centre François Baclesse, Caen, France: Pascaline Berthet, Laurent Castera, Dominique Vaur. Institut Paoli Calmettes, Marseille, France: Violaine Bourdon, Catherine Noguès, Tetsuro Noguchi, Cornel Popovici, Audrey Remenieras, Hagay Sobol. CHU Arnaud-de-Villeneuve, Montpellier, France: Isabelle Coupier, Pascal Pujol. Centre Oscar Lambret, Lille, France: Claude Adenis, Aurélie Dumont, Françoise Révillion. Centre Paul Strauss, Strasbourg, France: Danièle Muller. Institut Bergonié, Bordeaux, France: Emmanuelle Barouk-Simonet, Françoise Bonnet, Virginie Bubien, Michel Longy, Nicolas Sevenet, Institut Claudius Regaud, Toulouse, France: Laurence Gladieff, Rosine Guimbaud, Viviane Feillel, Christine Toulas. CHU Grenoble, France: Hélène Dreyfus, Christine Dominique Leroux, Magalie Peysselon, Rebischung. CHU Dijon, France: Amandine Baurand, Geoffrey Bertolone, Fanny Coron, Laurence Faivre, Caroline Jacquot, Sarab Lizard. CHU St-Etienne, France: Caroline Kientz, Marine Lebrun, Fabienne Prieur. Hôtel Dieu Centre Hospitalier, Chambéry, France: Sandra Fert Ferrer. Centre Antoine Lacassagne, Nice, France: Véronique Mari. CHU Limoges, France: Laurence Vénat-Bouvet. CHU Nantes, France: Stéphane Bézieau, Capucine Delnatte. CHU Bretonneau, Tours and Centre Hospitalier de Bourges France: Isabelle Mortemousque. Groupe Hospitalier Pitié-Salpétrière, Paris, France: Florence Coulet, Mathilde Warcoin. CHU Vandoeuvre-les-Nancy, France: Myriam Bronner, Johanna Sokolowska. CHU Besançon, France: Marie-Agnès Collonge-Rame, Alexandre Damette. CHU Poitiers, Centre Hospitalier d'Angoulême and Centre Hospitalier de Niort, France: Paul Gesta. Centre Hospitalier de La Rochelle : Hakima Lallaoui. CHU Nîmes Carémeau, France : Jean Chiesa. CHI Poissy, France: Denise Molina-Gomes. CHU Angers, France : Olivier Ingster; CHU de Martinique, France: Odile Bera; Mickaelle Rose; Ilse Coene and Brecht Crombez;

Alicia Tosar and Paula Diaque; Drs .Sofia Khan, Taru A. Muranen, Carl Blomqvist, Irja Erkkilä and Virpi Palola; The Hereditary Breast and Ovarian Cancer Research Group Netherlands (HEBON) consists of the following Collaborating Centers: Netherlands Cancer Institute (coordinating center), Amsterdam, NL: M.A. Rookus, F.B.L. Hogervorst, F.E. van Leeuwen, M.A. Adank, M.K. Schmidt, D.J. Jenner; Erasmus Medical Center, Rotterdam, NL: J.M. Collée, A.M.W. van den Ouweland, M.J. Hooning, I.A. Boere; Leiden University Medical Center, NL: C.J. van Asperen, P. Devilee, R.B. van der Luijt, T.C.T.E.F. van Cronenburg; Radboud University Nijmegen Medical Center, NL: M.R. Wevers, A.R. Mensenkamp; University Medical Center Utrecht, NL: M.G.E.M. Ausems, M.J. Koudijs; Amsterdam UMC, Univ of Amsterdam, NL: I. van de Beek; Amsterdam UMC, Vrije Universiteit Amsterdam, NL: K. van Engelen, J.J.P. Gille; Maastricht University Medical Center, NL: E.B. Gómez García, M.J. Blok, M. de Boer; University of Groningen, NL: L.P.V. Berger, A.H. van der Hout, M.J.E. Mourits, G.H. de Bock; The Netherlands Comprehensive Cancer Organisation (IKNL): S. Siesling, J. Verloop; The nationwide network and registry of histo- and cytopathology in The Netherlands (PALGA): E.C. van den Broek. HEBON thanks the study participants and the registration teams of IKNL and PALGA for part of the data collection; Hong Kong Sanatorium and Hospital; the Hungarian Breast and Ovarian Cancer Study Group members (Janos Papp, Aniko Bozsik, Timea Pocza, Zoltan Matrai, Miklos Kasler, Judit Franko, Maria Balogh, Gabriella Domokos, Judit Ferenczi, Department of Molecular Genetics, National Institute of Oncology, Budapest, Hungary) and the clinicians and patients for their contributions to this study; the Oncogenetics Group (VHIO) and the High Risk and Cancer Prevention Unit of the University Hospital Vall d'Hebron, Miguel Servet Progam (CP10/00617), and the Cellex Foundation for providing research facilities and equipment; the ICO Hereditary Cancer Program team led by Dr. Gabriel Capella; the ICO Hereditary Cancer Program team led by Dr. Gabriel Capella; Dr Martine Dumont for sample management and skillful assistance; Ana Peixoto, Catarina Santos and Pedro Pinto; members of the Center of Molecular Diagnosis, Oncogenetics Department and Molecular Oncology Research Center of Barretos Cancer Hospital; Heather Thorne, Eveline Niedermayr, all the kConFab research nurses and staff, the heads and staff of the Family Cancer Clinics, and the Clinical Follow Up Study (which has received funding from the NHMRC, the National Breast Cancer Foundation, Cancer Australia, and the National Institute of Health (USA)) for their contributions to this resource, and the many families who contribute to kConFab; the KOBRA Study Group; Csilla Szabo (National Human Genome Research Institute, National Institutes of Health, Bethesda, MD, USA); Lenka Foretova and Eva Machackova (Department of Cancer Epidemiology and Genetics, Masaryk Memorial Cancer Institute and MF MU, Brno, Czech Republic); and Michal Zikan, Petr Pohlreich and Zdenek Kleibl (Oncogynecologic Center and Department of Biochemistry and Experimental Oncology, First Faculty of Medicine, Charles University, Prague, Czech Republic); Anne Lincoln, Lauren Jacobs; the participants in Hereditary Breast/Ovarian Cancer Study and Breast Imaging Study for their selfless contributions to our research; the NICCC National Familial Cancer Consultation Service team led by Sara Dishon, the lab team led by Dr. Flavio Lejbkowicz, and the research field operations team led by Dr. Mila Pinchev; the investigators of the Australia New Zealand NRG Oncology group; members and participants in the Ontario Cancer Genetics Network; Leigha Senter, Kevin Sweet, Caroline Craven, Julia Cooper, Amber Aielts, and Michelle O'Conor; Yip Cheng Har, Nur Aishah Mohd Taib, Phuah Sze Yee, Norhashimah Hassan and all the research nurses, research assistants and doctors involved in the MyBrCa Study for assistance in patient recruitment, data collection and sample preparation, Philip Iau, Sng Jen-Hwei and Sharifah Nor Akmal for contributing samples from the Singapore Breast Cancer Study and the HUKM-HKL Study respectively; the Meirav Comprehensive breast cancer center team at the Sheba Medical Center; Christina Selkirk; Åke Borg, Håkan Olsson, Helena Jernström, Karin Henriksson, Katja

Harbst, Maria Soller, Ulf Kristoffersson; from Gothenburg Sahlgrenska University Hospital: Anna Öfverholm, Margareta Nordling, Per Karlsson, Zakaria Einbeigi; from Stockholm and Karolinska University Hospital: Anna von Wachenfeldt, Annelie Liljegren, Annika Lindblom, Brita Arver, Gisela Barbany Bustinza, Johanna Rantala; from Umeå University Hospital: Beatrice Melin, Christina Edwinsdotter Ardnor, Monica Emanuelsson; from Uppsala University: Hans Ehrencrona, Maritta Hellström Pigg, Richard Rosenquist; from Linköping University Hospital: Marie Stenmark-Askmalm, Sigrun Liedgren; Cecilia Zvocec, Qun Niu; Joyce Seldon and Lorna Kwan; Dr. Robert Nussbaum, Beth Crawford, Kate Loranger, Julie Mak, Nicola Stewart, Robin Lee, Amie Blanco and Peggy Conrad and Salina Chan; Simon Gayther, Susan Ramus, Paul Pharoah, Carole Pye, Patricia Harrington and Eva Wozniak; Geoffrey Lindeman, Marion Harris, Martin Delatycki, Sarah Sawyer, Rebecca Driessen, and Ella Thompson for performing all DNA amplification.
