# Supplementary material for: Modification of BRCA1-associated breast cancer risk by HMMR overexpression
Source: Nat Commun. 2022 Apr 7;13:1895. doi: 10.1038/s41467-022-29335-z (PMC8989921; doi:10.1038/s41467-022-29335-z)
Supplement: Supplementary file 3 — Description of Additional Supplementary Files [file 41467_2022_29335_MOESM3_ESM.pdf]

Description of Additional Supplementary Files

File Name: Supplementary Data 1
Description: Results of gene set-based analysis of GWAS summary statistics.

File Name: Supplementary Data 2
Description: Cancer keyword analysis of risk-associated genet sets.

File Name: Supplementary Data 3
Description: BRCA1 and risk-associated gene set correlations.

File Name: Supplementary Data 4
Description: Risk-linked gene sets including BRCA1 and/or HMMR.

File Name: Supplementary Data 5
Description: Risk-linked gene sets coexpressed with HMMR.

File Name: Supplementary Data 6
Description: Histopathological and marker features of mouse mammary tumors.
