# Supplementary material for: Modification of BRCA1-associated breast cancer risk by HMMR overexpression
Source: Nat Commun. 2022 Apr 7;13:1895. doi: 10.1038/s41467-022-29335-z (PMC8989921; doi:10.1038/s41467-022-29335-z)
Supplement: Supplementary file 10 — Reporting Summary [file 41467_2022_29335_MOESM10_ESM.pdf]

nature research

Corresponding author(s): MA Pujana, CA Maxwell

Last updated by author(s): Mar 5, 2022

Reporting Summary

Nature Research wishes to improve the reproducibility of the work that we publish. This form provides structure for consistency and transparency in reporting. For further information on Nature Research policies, see our Editorial Policies and the Editorial Policy Checklist.

Statistics

For all statistical analyses, confirm that the following items are present in the figure legend, table legend, main text, or Methods section.

n/a	Confirmed	
☐	☒	The exact sample size (*n*) for each experimental group/condition, given as a discrete number and unit of measurement
☐	☒	A statement on whether measurements were taken from distinct samples or whether the same sample was measured repeatedly
☐	☒	The statistical test(s) used AND whether they are one- or two-sided<br>*Only common tests should be described solely by name; describe more complex techniques in the Methods section.*
☐	☒	A description of all covariates tested
☐	☒	A description of any assumptions or corrections, such as tests of normality and adjustment for multiple comparisons
☐	☒	A full description of the statistical parameters including central tendency (e.g. means) or other basic estimates (e.g. regression coefficient) AND variation (e.g. standard deviation) or associated estimates of uncertainty (e.g. confidence intervals)
☐	☒	For null hypothesis testing, the test statistic (e.g. *F*, *t*, *r*) with confidence intervals, effect sizes, degrees of freedom and *P* value noted<br>*Give P values as exact values whenever suitable.*
☒	☐	For Bayesian analysis, information on the choice of priors and Markov chain Monte Carlo settings
☒	☐	For hierarchical and complex designs, identification of the appropriate level for tests and full reporting of outcomes
☐	☒	Estimates of effect sizes (e.g. Cohen's *d*, Pearson's *r*), indicating how they were calculated

Our web collection on statistics for biologists contains articles on many of the points above.

Software and code

Policy information about availability of computer code

Data collection	Fixed cells were imaged using Fluoview software (Olympus) connected to an Olympus Fluoview FV10i confocal microscope. Images were captured using a 60X-300X 1-2 NA oil objectives as a stack of 5-7 optical sections with a spacing of 0.5 microm through the cell volume. The images were analyzed using Fiji-ImageJ (National Institute of Health, version 1.52s 10) to generate maximum intensity projection of the fluorescence channels.<br><br>The cell living cultures were imaged with the ImageXpress Micro XL epifluorescence microscope (Molecular Devices Inc.) controlled by MetaXpress software (Molecular Devices Inc., version 5.0.2.0). Images were captured through a 40X 0.75 NA dry objective with 2x2 binned resolution. For blebbing analysis, images were taken every 1 minute. For F-actin-labeled live cell imaging of parental HeLa and Tet-On HeLa, the cells were incubated with CellLight Actin-RFP, BacMam 2.0 (C10583, Thermo Fisher Scientific) 48 hours before imaging, and Tet-On HeLa cells were treated with doxycycline 24 hours before imaging. Images were captured every 5 minutes.<br><br>Mass spectrometry. The Easy nLC ultra-high-pressure LC system (Thermo Fisher Scientific) was coupled to a Q Exactive HF mass spectrometer with an EASY-Spray source to perform liquid chromatography tandem mass spectrometry analysis.<br><br>Targeted sequencing was performed on an Ion Chef and Ion Torrent S5 platforms following manufacturer's protocols (Thermo Fisher Scientific). Basal data processing and quality control was performed using the AmpliSeqRNA plug-in for Ion Torrent S5. Differential gene expression analysis was performed in Transcriptome Analysis Console (version 4.0.1).
Data analysis	The pathway scoring algorithm (Pascal tool: https://www2.unil.ch/cbg/index.php?title=Pascal#:~:text=Pascal%20(Pathway%20scoring%20algorithm)%20is,for%20genes%20and%20predefined%20pathways.) was applied with default parameters considering genetic variants with minor allele frequency > 5% and using the gene-locus sum chi-squared test for significance assessment.

The scores were used to define edge length in undirected keyword networks constructed using the ggnetwork (version 0.5.10) and network (version 1.17.1) R packages.

Univariate survival analyses of rs299290 genotypes in TCGA breast cancer subtypes and of mammary mouse tumor incidence were performed using the Kaplan–Meier method and log-rank test, computed with the survival package (version 3.2-13) in R.

The tumor RNA-seq reads were trimmed for adaptors, masked for low-complexity and low-quality sequences, and subsequently quantified for transcript expression using Kallisto (version 0.43.16) and mouse genome version mm9. Gene-level quantification was carried out using the tximport (version 3.14) Bioconductor package, mm9 and Ensembl v94 annotations. Differential expression was analyzed using DESeq2 (version 2.13). GO term enrichment was analyzed using clusterProfiler (version 3.14) and GOnet (version 2019-07-01). The pre-ranked GSEA method (version 4.1) was applied based on standard parameters. Gene ontology terms for genes with differential expression identified from RNA-seq or TaqMan assays were identified with GOrilla (version 01-2019) and g:Profiler (version 0.2.1).

The gene set expression scores were computed using the single-sample GSEA (ssGSEA) algorithm calculated within the GSVA software (version 1.43.1), and using primary breast tumor gene expression RNA-seq (Fragments Per Kilobase of transcript per Million mapped reads upper quartile (FPKM-UQ)) TCGA data.

Immunohistochemistry quantifications were performed using Fiji-ImageJ (National Institute of Health, version 1.52s-10) software with the IHC profiler plugin. The immunofluorescence images were analyzed using Fiji-ImageJ.

Acquired spectra were searched using Proteome Discoverer software (Thermo Fisher Scientific, version 2.5) against the Homo sapiens reference proteome including isoforms, downloaded from the UniProt database74 (version 2017-07). Differential abundance in anti-HMMR versus rabbit IgG immunoprecipitates was determined using a Student's t-test and proteins commonly identified in affinity enrichment experiments were flagged based on information derived from the CRAPome (version 2.0) database.

The R-code used to compute the weighted score of literature keywords and to construct undirected networks is available at GitHub (https://github.com/pujana-lab/HMMR).

For manuscripts utilizing custom algorithms or software that are central to the research but not yet described in published literature, software must be made available to editors and reviewers. We strongly encourage code deposition in a community repository (e.g. GitHub). See the Nature Research guidelines for submitting code & software for further information.

Data

Policy information about availability of data

All manuscripts must include a data availability statement. This statement should provide the following information, where applicable:

- Accession codes, unique identifiers, or web links for publicly available datasets
- A list of figures that have associated raw data
- A description of any restrictions on data availability

The RNA-sequencing data generated in this study have been deposited in the Gene Expression Omnibus database under accession number GSE163756 (preneoplastic mammary tissue; https://www.ncbi.nlm.nih.gov/geo/query/acc.cgi?acc=GSE163756) and GSE164004 (mammary tumors; https://www.ncbi.nlm.nih.gov/geo/query/acc.cgi?acc=GSE164004). The raw and processed data IP-MS data generated in this study have been deposited in the Proteome Exchange (PXD031752: http://proteomecentral.proteomexchange.org/cgi/GetDataset?ID=PXD031752) and MassIVE (MSV000088870: doi:10.25345/C57659F06) repositories. The publicly available GWAS data used in this study are available in the CIMBA consortium web page (https://cimba.ccge.medschl.cam.ac.uk/projects/) and the publicly available single-cell Brca1-associated mouse mammary tumorigenesis data used in this study are available in the ArrayExpress database under accession code E-MTAB-10043 (https://www.ebi.ac.uk/arrayexpress/experiments/E-MTAB-10043/; processed data is also available in http://marionilab.cruk.cam.ac.uk/BRCA1Tumourigenesis). The remaining data are available within the Article, Supplementary Information or Source Data file.

Field-specific reporting

Please select the one below that is the best fit for your research. If you are not sure, read the appropriate sections before making your selection.

[x] Life sciences [ ] Behavioural & social sciences [ ] Ecological, evolutionary & environmental sciences

For a reference copy of the document with all sections, see nature.com/documents/nr-reporting-summary-flat.pdf

Life sciences study design

All studies must disclose on these points even when the disclosure is negative.

Sample size	Power studies were applied to determine sample size. Specifically, mice studies were evaluated using a two-sided test with a 5% significance level to predict a 85-90% power to reject the null hypothesis of no difference between genotype groups. For gene expression, molecular and cellular assays, no specific sample-size calculations were performed: sample sizes were chosen to provide enough replicates for statistical analysis and were determined by availability of biological samples and/or based on experience with the biological models.
Data exclusions	No exclusions

Replication	The number of replicas in each experiment, and the number of independent experiments are detailed for each analysis (in each corresponding figure legend). All molecular and cellular assays were performed at least 2 times (including 2-4 replicates) with similar trends observed.
Randomization	Three (mice studies, genotype-groups) and sample (molecular and cellular assays) groups were compared with no randomization. Randomization was not applicable because defined genotype/sample sets were compared.
Blinding	Tissue sample collection were not blind relative to mouse group allocation. Analysis of tumor features and immunohistochemistry results were blind relative to group allocation. Molecular and gene expression analyses were not blind relative to group allocation.

Reporting for specific materials, systems and methods

We require information from authors about some types of materials, experimental systems and methods used in many studies. Here, indicate whether each material, system or method listed is relevant to your study. If you are not sure if a list item applies to your research, read the appropriate section before selecting a response.

Materials & experimental systems

n/a	Involved in the study
☐	☒ Antibodies
☐	☒ Eukaryotic cell lines
☒	☐ Palaeontology and archaeology
☐	☒ Animals and other organisms
☐	☒ Human research participants
☒	☐ Clinical data
☒	☐ Dual use research of concern

Methods

n/a	Involved in the study
☒	☐ ChIP-seq
☒	☐ Flow cytometry
☒	☐ MRI-based neuroimaging

Antibodies

Antibodies used	Antibodies used in this study are detailed in Supplementary Table 9 (Supplementary file), including vendor, method and dilution.
Validation	All antibodies are validated according to manufacturer information. No custom antibodies were used in this study.

Eukaryotic cell lines

Policy information about cell lines

Cell line source(s)	HeLa cells were purchased from the American Type Culture Collection (ATCC, catalog CCL-2TM) and cultured in 10% FBS DMEM, 20 U/ml penicillin (Invitrogen) and 20 microg/ml streptomycin (Invitrogen). HEK-293FT cells were obtained from Invitrogen (Thermo Fisher, catalog R70007). The 4T1 and MCF7 cell lines were obtained from ATCC (CRL-2539TM and HTB-22TM, respectively) and were grown according to recommendations.
Authentication	Authenticated by ATCC and Thermo Fisher. The four cell lines were purchased for this study.
Mycoplasma contamination	All cell lines and cultures used in this study were tested every 1-2 weeks through the whole study. The cell lines were tested negative before each assay included in the study.
Commonly misidentified lines (See ICLAC register)	No commonly misidentified cell lines were used in this study.

Animals and other organisms

Policy information about studies involving animals; ARRIVE guidelines recommended for reporting animal research

Laboratory animals	For this study we purchased C57BL/6 (Charles River) and Trp53tm1Brd Brca1tm1Aash/F22-24 Tg(LGB-cre)74Acl/J mouse strain (catalog 012620, The Jackson Laboratory). Female mice were monitored every 2-3 days for tumor development and euthanized when incident tumors were detected or at the end of the study (no tumor), maximum 50 weeks after second pregnancy; tumor sizes were < 2000 mm3. Total time follow-up per female mouse: < 80 weeks.
Wild animals	No wild animals were used in this study.
Field-collected samples	No field-collected samples were used in this study.
Ethics oversight	All animal experiments were carried out in the University of Barcelona-Bellvitge animal facility, under the Generalitat de Catalunya license authority (reference 9774) and approval of the IDIBELL University of Barcelona-Bellvitge Ethics Committee.

Note that full information on the approval of the study protocol must also be provided in the manuscript.

Human research participants

Policy information about studies involving human research participants

Population characteristics	The GWAS summary statistics of BRCA1-associated and triple-negative breast cancer were downloaded from the public repository of the Consortium of Investigators of Modifiers of BRCA1/2 (CIMBA: http://cimba.ccge.medschl.cam.ac.uk/projects/). This study did not require individual data.<br><br>Highly purified subpopulations of human mammary epithelial cells were isolated from normal reduction mammoplasty tissue samples from premenopausal women non-carriers and carriers of BRCA1 pathological variants. The breast tissue were confirmed to be histologically normal. The donors were not on preventive therapy.
Recruitment	The donors were recruited randomly among premenopausal women non-carriers and carriers of BRCA1 pathological variants.
Ethics oversight	The study of normal breast tissue from women non-carriers and carriers of BRCA1 pathological variants was approved by the University of British Columbia's Ethics Review Board (reference H19-04034). All donors provided written informed consent.

Note that full information on the approval of the study protocol must also be provided in the manuscript.
